# As Blind as a Bat? Opsin Phylogenetics Illuminates the Evolution of Color Vision in Bats

**DOI:** 10.1093/molbev/msy192

**Published:** 2018-11-23

**Authors:** Bruno F Simões, Nicole M Foley, Graham M Hughes, Huabin Zhao, Shuyi Zhang, Stephen J Rossiter, Emma C Teeling

**Affiliations:** 1 UCD School of Biology and Environmental Science, University College Dublin, Dublin 4, Ireland; 2 School of Earth Science, University of Bristol, Bristol, United Kingdom; 3 School of Biological Science, The University of Adelaide, South Australia, Australia; 4 Department of Ecology and Hubei Key Laboratory of Cell Homeostasis, College of Life Sciences, Wuhan University, Wuhan 430072, China; 5 College of Animal Science and Veterinary Medicine, Shenyang Agricultural University, Shenyang, China; 6 School of Biological and Chemical Sciences, Queen Mary University of London, London, United Kingdom

**Keywords:** photic adaptation, color vision, sensory trade-off, visual pigments, bats

## Abstract

Through their unique use of sophisticated laryngeal echolocation bats are considered sensory specialists amongst mammals and represent an excellent model in which to explore sensory perception. Although several studies have shown that the evolution of vision is linked to ecological niche adaptation in other mammalian lineages, this has not yet been fully explored in bats. Recent molecular analysis of the opsin genes, which encode the photosensitive pigments underpinning color vision, have implicated high-duty cycle (HDC) echolocation and the adoption of cave roosting habits in the degeneration of color vision in bats. However, insufficient sampling of relevant taxa has hindered definitive testing of these hypotheses. To address this, novel sequence data was generated for the *SWS1* and *MWS/LWS* opsin genes and combined with existing data to comprehensively sample species representing diverse echolocation types and niches (*SWS1 n* = 115; *MWS/LWS n* = 45). A combination of phylogenetic analysis, ancestral state reconstruction, and selective pressure analyses were used to reconstruct the evolution of these visual pigments in bats and revealed that although both genes are evolving under purifying selection in bats, *MWS/LWS* is highly conserved but *SWS1* is highly variable. Spectral tuning analyses revealed that *MWS/LWS* opsin is tuned to a long wavelength, 555–560 nm in the bat ancestor and the majority of extant taxa. The presence of UV vision in bats is supported by our spectral tuning analysis, but phylogenetic analyses demonstrated that the *SWS1* opsin gene has undergone pseudogenization in several lineages. We do not find support for a link between the evolution of HDC echolocation and the pseudogenization of the *SWS1* gene in bats, instead we show the *SWS1* opsin is functional in the HDC echolocator, *Pteronotus parnellii*. Pseudogenization of the *SWS1* is correlated with cave roosting habits in the majority of pteropodid species. Together these results demonstrate that the loss of UV vision in bats is more widespread than was previously considered and further elucidate the role of ecological niche specialization in the evolution of vision in bats.

## Introduction

Bats possess some of the most unique and peculiar adaptations observed amongst extant mammals that render them as excellent models in which to study the evolution of sensory perception. They are successful, nocturnal animals (>1260 species), the only mammals that can truly fly (Simmons 2005) and they exist in diverse ecological niches throughout the globe, feeding on insects, small mammals, fish, blood, nectar, fruit, and pollen (Teeling et al. 2018). Bats are the only mammals to use laryngeal echolocation (Teeling et al. 2005; Teeling 2009; Teeling, Jones, and Rossiter 2016) for hunting, avoiding obstacles, and orienting in scotopic or low light conditions. This unique auditory capability shows great variation amongst the 21 families of echolocating bats (Teeling, Jones, and Rossiter 2016; Teeling et al. 2018) and appears to be related to lineage-specific selection pressures as well as shared ancestry (Teeling, Jones, and Rossiter 2016). It has been argued that bats have developed this acoustic sense at the expense of their other senses, such as vision, given the typically small size of an echolocating bat’s eyes (Eklöf et al. 2014) and has led to the popular use of the term “as blind as a bat.” One family of bats, the Pteropodidae (∼186 species), does not use laryngeal echolocation but instead has large sensitive eyes, specialized for nocturnal vision. 

In vertebrates, photosensitive molecules within the outer segments of rods and cones of the retina determine the spectral sensitivity of the eye. Each photosensitive molecule is encoded by a distinct opsin gene (Yokoyama and Radlwimmer 1998). Rods function in dim light and contain the rhodopsin pigment, which is most sensitive between 475 and 505 nm (e.g., Yokoyama 2008; Nathans et al. 1986). Cones are responsible for color vision and mammals can possess up to two types of opsin molecules with different sensitivities ranging from ∼360 to 440 nm (short-wavelength opsin; *SWS1*) and ∼536 to 560 nm (medium-to-long-wavelength opsin; *MWS/LWS*) (Yokoyama and Yokoyama 1996). Comparisons of visual pigments across taxa indicate that spectral tuning and, therefore, the wavelength of peak light sensitivity (*λ*_max_), is generally modulated by 5 key critical amino acid sites in *MWS/LWS* opsins (Yokoyama and Radlwimmer 1998) and at least 11 amino acid sites in *SWS1* opsin (Yokoyama et al. 2006). Therefore, although the five-site rule may be subject to allelic variation such as that observed in guppys (Kawamura et al. 2016) or may be reduced to a “three-site” rule in certain primates (Matsumoto et al. 2014), it is possible to infer the *λ*_max_ from opsin sequences using sequence based analyses. In this way, the spectral type of the *SWS1* (UV: ∼360 nm or visible: >400 nm) can be broadly determined, whereas *λ*_max_ predictions for the *MWS/LWS* are typically more accurate (Hauser, van Hazel, and Chang 2014). 

The evolution of the visual opsins is tightly correlated with ecological niche adaptation (Douglas and Jeffery 2014). The *MWS/LWS* is remarkably conserved across most mammals (Bowmaker and Hunt 2006), but recent studies suggest that the *MWS/LWS* opsin gene has undergone pseudogenization in some cetaceans, possibly coincident with the loss of the *SWS1* opsin gene (Meredith et al. 2013; Emerling et al. 2015; Springer et al. 2016). This adaptation is thought to be driven by inhabiting a deep-water environment and feeding on bioluminescent prey (Meredith et al. 2013; Springer et al. 2016). Adaptation to a fossorial niche is hypothesized to have driven the loss of both cone opsins (*SWS1* and *MWS/LWS*) in xenarthrans (Emerling and Springer 2015), the naked-mole rat (Kim et al. 2011), and the star-nosed and golden moles (Emerling and Springer 2014, 2015). Similar adaptations to burrowing habitats have been observed in fossorial snakes (Simões et al. 2015, 2016), caecilians (Mohun et al. 2010), and cave salamanders (Kos et al. 2001). Zhao, Rossiter, et al. (2009) showed that the *MWS/LWS* gene was conserved in all bat species but showed the *SWS1* gene had undergone dramatic divergent selection among lineages. This and subsequent analyses have shown that the *SWS1* opsin is functional in the majority of bat lineages and is sensitive to UV light (Zhao, Rossiter, et al. 2009; Emerling et al. 2015). These studies have also suggested that loss-of-function mutations in the *SWS1* has potentially coincided with the acquisition of high-duty cycle (HDC) echolocation, as well as with changes in roosting ecology in some lineages (e.g., cave roosting in Pteropodidae; Zhao, Rossiter, et al. 2009), however, incomplete sampling has hampered definitive testing of these hypotheses.

The loss of the *SWS1* is notably widespread across mammals, especially in lineages living in low light environments (Hunt et al. 1998; Bowmaker and Hunt 2006; Jacobs 2013; Meredith et al. 2013; Davies et al. 2015; Emerling et al. 2015; Wu, Wang, and Hadly 2017). Therefore, it has been suggested that the loss of the *SWS1* opsin is a nonadaptive process that has been tolerated by most mammals given that the interaction between the rhodopsin and *MWS/LWS* opsin still allows some color discrimination (Griebel and Schmid 1992). Increased interaction with other sensory systems (e.g., olfaction, hearing) may also reduce the impact of *SWS1* opsin loss on species’ survival (Zhao, Rossiter, et al. 2009; Fujun et al. 2012; Jacobs 2013). Furthermore, spectral shifts in UV sensitivity to violet/blue vision has occurred at least 12 times during mammalian evolution (Emerling et al. 2015). Given the correlation between opsin evolution and ecological niche specialization, the functionality and evolution of opsin genes has been used as a proxy to infer the ecology of ancestral lineages (Tan et al. 2005). Therefore, elucidating the evolution and functionality of cone visual pigments in ecologically divergent taxa can shed light on how vision has contributed towards mammalian adaptation to diverse ecological niches and also further our understanding of the evolutionary history of mammals.

Here, we have gathered, amplified, and sequenced the most extensive data set of opsin genes across the largest taxonomic representation of bat species (*n* = 115) to date. Our data set, which combines existing and novel sequence data, encompasses the vast diversity of ecological niches and acquisition of unique senses (visual, olfaction, thermal, and acoustic) across ∼65 million years of bat evolutionary history (Miller-Butterworth et al. 2007; Meredith et al. 2011; Foley et al. 2015; Foley, Springer, and Teeling 2016; Teeling et al. 2018). We used selection estimates (dN/dS ratios or *ω*) to elucidate the evolutionary forces acting on opsin genes (relaxed, neutral, or positive selection) in bats and assessed their influence on the spectral tuning of these visual pigments. Using comprehensive taxonomic sampling we determined if the loss of *SWS1* is always coincident with (1) the evolution of HDC echolocation in echolocating bats and (2) cave roosting in pteropodids. Furthermore, molecular clock dating, selection tests, and analysis of substitution rates were used to elucidate the timing of the *SWS1* pseudogenization event and aid our understanding of the selective pressures that have potentially driven these events in bats.

## Results

The *SWS1* data set of 115 bats species resulted in a coding alignment of 1125–1325 bp. The *MWS/LWS* data set contained 45 bat species and resulted in a coding alignment of 536 bp. Where applicable, *COX1* barcoding was used to confirm each species identity. In instances where the species was not present in the BOLD database a cut-off of =<90% sequence identity was used to identify samples to genus level.

### *MWS/LWS* Functionality, Sensitivity, and Phylogenetics

**Fig. 1.**
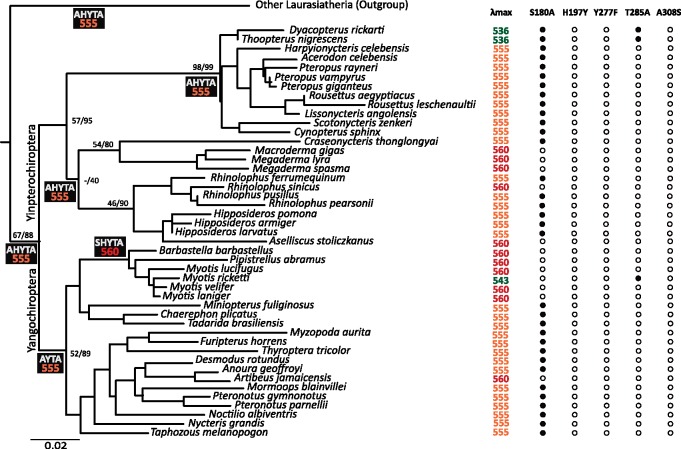
Ancestral state reconstruction spectral tuning sites and inferred *λ*_max_ of the MWS/LWS opsin gene for major bat clades. Inferred *λ*_max_ values are shown for each species in addition to the observed changes in the five MWS/LWS spectral sites for each species. The spectral sites for the MWS/LWS opsin and estimated *λ*_max_ for the laurasiatherian ancestor is based on Liu et al. (2018).

The *MWS/LWS* opsin gene was found to be functional and highly conserved across all bats, with no indels, frameshifts, or apparent alternative splice sites. Phylogenetic reconstructions conducted using maximum likelihood (ML) and Bayesian analyses (BA) recovered the consensus bat species tree (fig. 1) (Teeling et al. 2000, 2002, 2005; Miller-Butterworth et al. 2007; Meredith et al. 2011; Foley et al. 2015). ML bootstrap support (BS) was low across the tree (fig. 1) whereas Bayesian posterior probabilities (BPP) were higher throughout. Ancestral state reconstruction and analyses of the spectral tuning sites (*λ*_max_) exhibited high levels of conservation throughout bats (fig. 1). The majority of extant bat species (*n* = 31) and the putative crown-group ancestor have a substitution in the first spectral tuning site, S180A (S→A at site 180), present in eutherian and laurasiatherian ancestors (Liu et al. 2018) which differs from the ancestral vertebrate condition of “SHYTA” (∼560 nm; Yokoyama, Yang, and Starmer 2008), and indicates sensitivity to wavelengths of ∼555 nm (fig. 1). The ancestral eutherian spectral pattern (“SHYTA”) (Yokoyama, Yang, and Starmer 2008; Liu et al. 2018) is observed in the Megadermatidae, *Rhinolophus sinicus*, the Vespertilionidae (except *Myotis ricketii*), and *Artibeus jamaicensis*. A medium-wavelength shift resulting from a mutation in the fourth spectral site, T285A is observed in *Myotis ricketti* and reduces the spectral sensitivity to 543 nm as independently reported by Zhao, Rossiter, et al. (2009). A combination of both aforementioned spectral site mutations are present in two cave dwelling pteropodid bats, *Dyacopterus rickarti* and *Thoopterus nigrescens* and reduces the spectral sensitivity to ∼536 nm (fig. 1).

### Functionality and Spectral Tuning of the *SWS1* Opsin

**Fig. 2.**
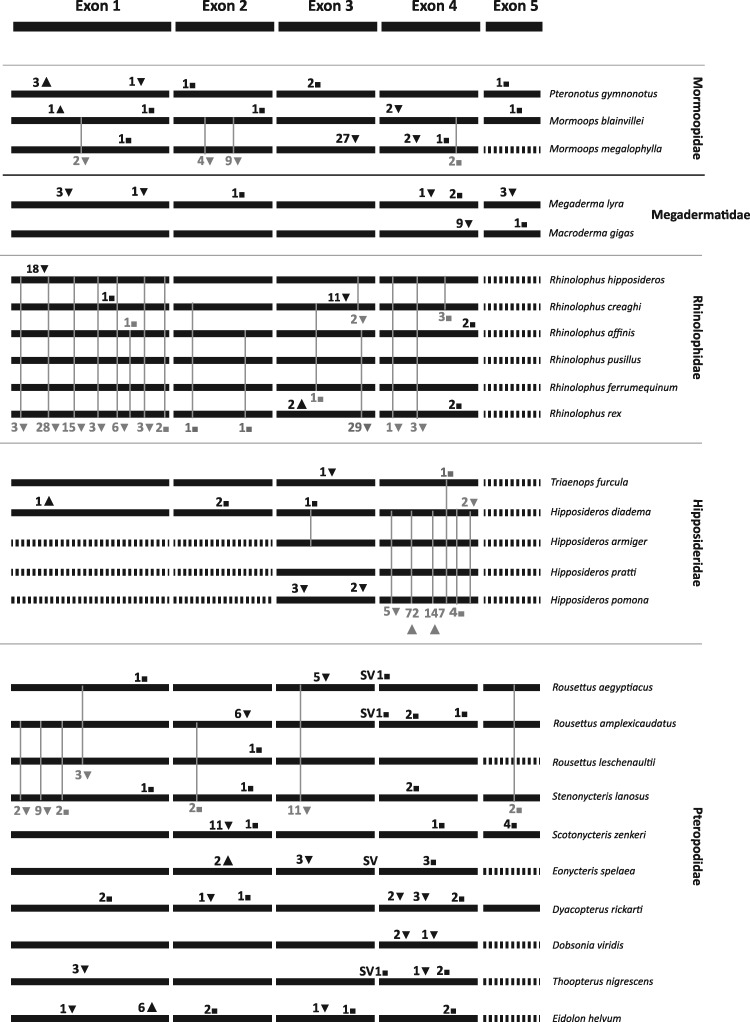
Schematic depicting the shared and private indels leading to frame-shift mutations (insertion ▲, deletion▼), premature stop codons (◼) and loss-of-function mutations in the SWS1 opsin in 26 bats where this gene is nonfunctional.

**Fig. 3.**
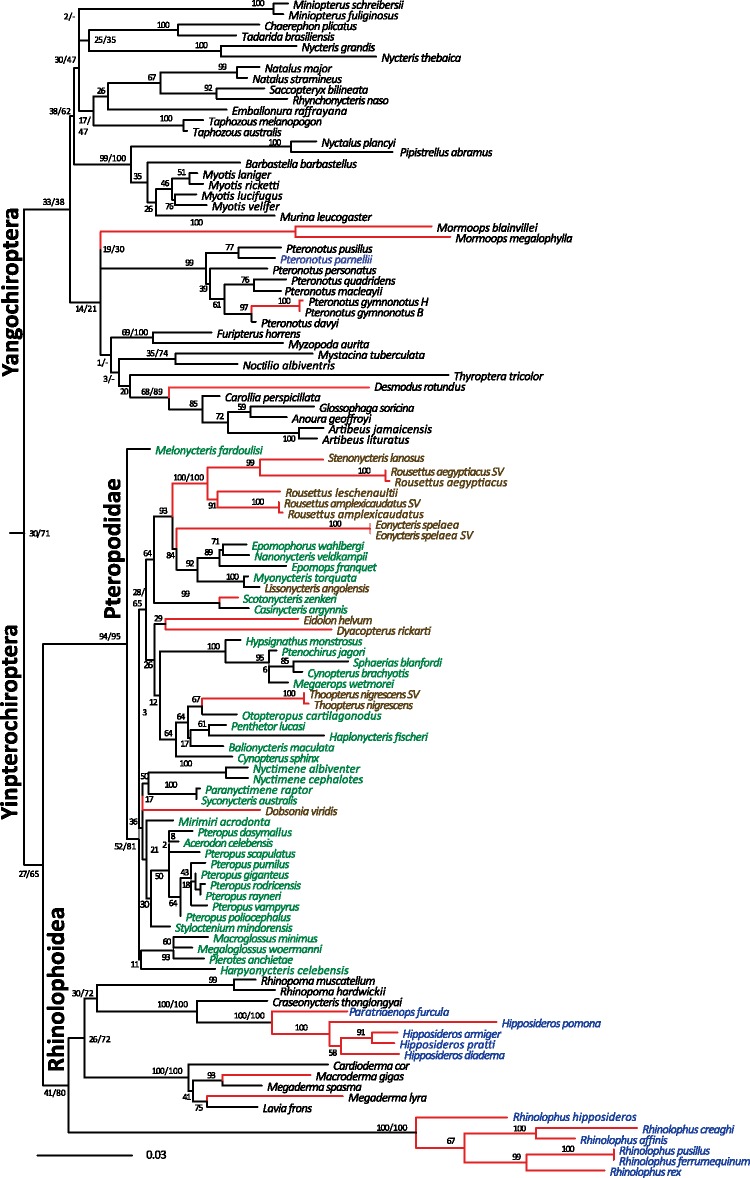
Phylogram inferred from ML analysis of the *SWS1* opsin gene. Nodal support for both the ML and BA analysis are shown at nodes corresponding to major clades. Species using HDC echolocation are highlighted in blue. Red branches correspond to tips in which the *SWS1* gene is nonfunctional. The Pteropodidae are highlighted in green, where brown is used to denote cave-dwelling taxa.

Unlike the *MWS/LWS* gene, the *SWS1* opsin gene is highly variable across bats. Multiple indels leading to frame-shift mutations, premature stop codons, and loss-of-function mutations were found in the major bat lineages of the Yinpterochiroptera (Pteropodidae, Rhinolophidae, Hipposideridae, Rhinonycteridae, and Megadermatidae), and the Yangochiroptera lineage Mormoopidae (figs. 2 and 3). Of the 115 bat species examined, the *SWS1* opsin appears to be nonfunctional in 26 species (figs. 2 and 3). In the suborder Yangochiroptera, loss-of-function mutations can be found exclusively in the New World family Mormoopidae. The genus *Mormoops* shares two ORF-disrupting deletions in the first and second exon. *Pteronotus gymnonotus* contains a single base pair deletion leading to a premature stop codon in exon 1. This deletion was confirmed through PCR and sequencing repeated in two different laboratories with two different specimens. No ORF disrupting mutations were observed in *Pteronotus parnellii*, suggesting the presence of a functional *SWS1* protein in this species.

In the superfamily Rhinolophoidea, ORF disrupting indels can be found in the Rhinolophidae (genus *Rhinolophus*), Hipposideridae (genus *Hipposideros*), and Rhinonycteridae (genus *Paratriaenops*) which all use HDC echolocation (figs. 2 and 3). Shared indels are found within all three families (fig. 2) but not between Rhinolophidae and Hipposideridae + Rhinonycteridae, suggesting that the loss-of-function events in these families, for the most part, postdate the divergence of each lineage (Foley et al. 2015). Additionally, independent ORF-disrupting mutations can be found in 2 of the 5 species from the family Megadermatidae (*Megaderma lyra* and *Macroderma gigas*), however within *Macroderma gigas*, the premature stop codon is only present in exon 5 (fig. 2). In the Pteropodidae, a shared ORF-disrupting mutation is present within the genus *Rousettus* and independent frameshift indels can be found in *Eonycteris spelaea, Dobsonia viridis, Thoopterus nigrescens*, and *Eidolon helvum* (fig. 2). An 11 bp deletion in exon 2 with premature stop codons was found in the tree roosting bat *Scotonycteris zenkeri* (fig. 2). All other pteropodid bats, including the cave roosting *Lissonycteris angolensis*, were shown to have a functional and intact *SWS1* opsin gene. The associations between loss of *SWS1* opsin gene and acquisition of primarily cave-roosting ecologies are supported by the estimation of contrasts using the Brunch algorithm (supplementary table S5, Supplementary Material online).

The intron–exon boundaries were found to be conserved across all echolocating bats and all tree roosting pteropodid bats, retaining the GT/AG pattern. Potential alternative splice sites were found in *Rousettus aegyptiacus* as well as *Rousettus amplexicaudatus*, *Eonycteris spelaea*, and *Thoopterus nigrescens* as was reported in previous studies (Shi, Radlwimmer, and Yokoyama 2001; Zhao, Rossiter, et al. 2009). An investigation of the 11 amino acid sites responsible for the spectral tuning of the *SWS1* visual pigment revealed that for all bat species analyzed the *SWS1* opsin is UV sensitive. The amino acid positions 86, 93, and 113 (bovine rhodopsin numbering) play a major role in the tuning of the vertebrate *SWS1* visual pigments to UV (Shi, Radlwimmer, and Yokoyama 2001; Yokoyama, Yang, and Starmer 2008) with site 86 being the largest contributor. All of these spectral sites are highly conserved across bats. Independent of method used, the ancestral reconstruction of the *SWS1* opsin spectral sites for all major lineages indicated that the bat ancestral *SWS1* opsin was sensitive to UV, with a phenylalanine present at site 86 in all extant and ancestral lineages. This suggests that no major changes occurred in bats’ spectral sensitivity prior to multiple losses of the *SWS1* in select bat lineages.

### *SWS1* Opsin Phylogenetics

ML and BA of the *SWS1* complete data sets support the consensus phylogenetic position of the majority of bat families and the monophyly of the two suborders Yangochiroptera and Yinpterochiroptera (fig. 3). However, the ML BS and BPP support across the topology of the tree were low (BS 30 and BPP 71, for the Yangochiroptera/Yinpterochiroptera split, fig. 3). The branch lengths in the *SWS1* opsin tree vary greatly across lineages and are particularly long in nonfunctional branches due to the large numbers of indels/stop codons in Rhinolophidae, Hipposideridae, Rhinonycteridae, and *Mormoops* (fig. 3).

Phylogenetic analyses of the functional *SWS1* data unite the laryngeal echolocating bats (supplementary fig. S1, Supplementary Material online). Alternative topology tests were used to compare phylogenetic support for the gene tree (fig. 3) topology versus species tree topology (Teeling, Jones, and Rossiter 2016) from our data. Alternative topology tests could not reject either topology (SH = 0.811 and SH = 0.189, respectively). Convergence between echolocating bats may be due to nonneutral convergent amino acid evolution between the Rhinolophoidea and Yangochiroptera. When this was tested, it was found that among 2 branch pairs examined, nine convergent amino acid sites, which included one spectral site F47L (F46L in the bovine rhodopsin), drives the convergent signal uniting echolocating bats in a single clade.

### dN/dS Analyses in the MWS/LWS Opsin Gene

The *ω* (dN/dS ratio) estimates across all branches and all sites for the *MWS/LWS* opsin gene was 0.0827, indicating that the *MWS/LWS* opsin gene is evolving under strong purifying selection (table 1). The *ω* estimates for each branch of the tree were consistently <1 (0.00–0.61) across all bat lineages. The two-ratio model for lineages with HDC echolocation had a higher *ω* value (*ω*  =  0.1504) than the background (*ω*  =  0.0736) (table 1). Conversely, Yinpterochiroptera and nonecholocating bats have higher *ω* estimates (0.0943 and 0.0940, respectively) than the backgrounds branches to which they were compared (0.0759 and 0.0831, respectively). The *ω* values are lower for cave roosting bats and insectivore/carnivore bats (0.081 and 0.082, respectively) than the background branches (0.090 and 0.086, respectively). The site models M8 β&ω and M2a detected four individual sites under positive selection under Bayes empirical Bayes analysis (supplementary table S4, Supplementary Material online). Both models detected positive selection in one of the five amino acid positions involved in the spectral tuning of the *MWS/LWS* opsin, S180A, located in the transmembrane domain 4. The FUBAR analysis indicated that this site is evolving under pervasive diversifying selection. The other sites under positive selection are located in transmembrane domains 4 and 5 and one in the extracellular loop 4. Episodic selection (selection affecting only a subset of lineages) was detected in only one lineage, the cave-roosting pteropodid *Lissonycteris angolensis* (*ω*_mean_ = 0.64, *P* < 0.005).

**Table 1.** *ω* Values and LRTs of Selective Pressures in the Bat MWS/LWS Opsin Gene.

Models	*ω* (dN/dS)	ln *L*^a^	NP^b^	Models Compared	2Δ^c^	*P*-value
MWS/LWS opsin gene						
1. One ratio	*ω* = 0.0827	3,200.90	88			
2. Free ratio	Variable by branch	3,136.94	173	1 vs. 2	127.9	0.002
3. M1a	*p*1 = 0.9465; *p*2 = 0.0535	3,128.68	89			
	*ω*1 = 0.040; *ω*2 = 1					
4. M2a	*p*1 = 0.9465; *p*2 = 0.0281; *p*3 = 0.0254	3,128.68	91	3 vs. 4	0	1
	*ω*1 = 0.0402; *ω*2 = 1; *ω*3 = 1					
5. M7β	*p* = 0.1495; *q* = 1.2597;	3,136.69	89			
	*ω* = 0.1020					
6. M8 β&ω	*p*0 = 0.9624; *p*1 = 0.4673;	3,125.14	91	5 vs. 6	23.1	9.64^−06^
	*q* = 9.1088 (*p*2 = 0.0376)					
	*ω* = 1.1077					
7. Two ratio (*ω*1, SWS1 functional; *ω*2, SWS1 nonfunctional	*ω*1 = 0.1194; *ω*2 = 0.0710	3,256.47	89	1 vs. 7	111.1	5.51^−26^
8. Two ratio (*ω*1, HDC; *ω*2, LDC	*ω*1 = 0.1504; *ω*2 = 0.0736	3,248.89	89	1 vs. 8	95.9	1.20^−22^
9. Two ratio (*ω*1, Yinpterochiroptera; *ω*2, Yangochiroptera	*ω*1 = 0.0943; *ω*2 = 0.0759	3,258.46	89	1 vs. 9	115.1	7.40^−27^
10. Two ratio (*ω*1, nonecholocating; *ω*2, echolocating)	*ω*2 = 0.0940; *ω*1 = 0.0831	3,258.85	89	1 vs. 10	115.9	4.99^−27^
11. Two ratio (*ω*1, primarily cave roosters; *ω*2, Other)	*ω*1 = 0.081; *ω*2 = 0.090	3,259.15	89	1 vs. 11	116.5	3.69^−27^
12. Two ratio (*ω*1, insectivore/carnivore; *ω*2, frugivore/nectivore)	*ω*1 = 0.082 ; *ω*2 = 0.086	−3,258.93	89	1 vs. 12	116.06	4.61^−27^

a The log likelihood values.

b Number of parameters.

c 2Δ = 2 × (ln *L*1 – ln *L*2).

### dN/dS Analyses in the SWS1 Opsin Gene

The estimates of *ω* values for the *SWS1* opsin on all branches and all sites was 0.1947, which is significantly smaller than 1, suggesting that the *SWS1* opsin gene is also evolving under purifying selection in bats (table 2). The free ratio model for all bats fits the data significantly better than the simple one-ratio model. This finding, together with results from multiple two-ratio models, in which the foreground branch is allowed to have a different ratio from the rest of the tree (background), suggests that different selective pressures may be acting along one or more lineages. Results from the two-ratio models confirm that *ω* values in lineages where *SWS1* is expected to be nonfunctional (*ω*  =  0.511) are higher than branches where *SWS1* is functional (*ω*  =  0.111). In addition, the two-ratio analysis showed that lineages with HDC echolocation had a higher *ω* value (*ω*  =  0.4646) compared with background branches (*ω*  =  0.1638). Furthermore, *ω* estimates were higher in the Yinpterochiroptera (*ω*  =  0.2448) than the Yangochiroptera (*ω*  =  0.1334). Similar differences in the *SWS1* between Yinpterochiroptera and Yangochiroptera can be found if we remove the pseudogenes (0.116 vs. 0.092, *P* = 9.39^−48^). This analysis also showed that nonecholocating bats (Pteropodidae) had a higher *ω* value (0.2047; *n* = 46) than echolocating bats (0.1836; *n* = 69). The *ω* values are also higher in primarily cave roosting bat species (*ω*  =  0.2957, *n* = 53) than bats that roost in trees or other roosting sites (*ω* =  0.1219, *n* = 62). The *ω* values are similar between species that feed on insects and other animals (*ω* =  0.1845, *n* = 56) and animals that feed on fruits and nectar (*ω* =  0.2014, *n* = 59). The site models M8 β&ω and M2a (BEB) detected 5 and 1 individual sites under positive selection, respectively across the entire data set (supplementary table S4, Supplementary Material online). Further examination showed that none of these sites are involved in the spectral sensitivity of the visual pigment and all are located in extra- and intra-cellular loops of the *SWS1* opsin. Episodic directional selection was detected in the *SWS1* opsin gene in *Pteronotus gymnonotus* (*ω*_mean_ = 0.56, *P* < 0.005), in the Rhinolophidae (*ω*_mean_ = 0.69, *P* < 0.005), and within this family in *Rhinolophus rex* (*ω*_mean_ = 0.64, *P* < 0.005).

**Table 2.** *ω* Values and LRTs of Selective Pressures in the Bat *SWS1* Opsin Gene in Chiroptera and Pteropodidae.

Models	*ω* (dN/dS)	ln *L*^a^	NP^b^	Models Compared	2Δ^c^	*P*-value
*SWS1* opsin gene: Chiroptera						
1. One ratio	*ω* = 0.1947	−9,713.82	220			
2. Free ratio	Variable by branch	−9,577.64	437	1 vs. 2	489.9	3.83^−23^
3. M1a	*p*1 = 0.8021; *p*2 = 0.19790	−9,529.13	221			
	*ω*1 = 0.1127; *ω*2 = 1					
4. M2a	*p*1 = 0.7978; *p*2 = 0.1313; *p*3 = 0.0709	−9,529.13	223	3 vs. 4	0	1
	*ω*1 = 0.1116; *ω*1 = 1; *ω*2 = 1					
5. M7β	*p* = 0.5843; *q* = 1.7967	−9,577.89	221			
6. M8 β&ω	*p*0 = 0.9958; *p*1 = 0.6198;	−9,565.24	222	5 vs. 6	25.3	3.21^−06^
	*q* = 2.0180 (*p*2 = 0.0042);					
	*ω* = 4.0557					
7. Two ratio (*ω*1, functional; *ω*2, nonfunctional	*ω*1 = 0.111; *ω*2 = 0.5116	−9,775.90	221	1 vs. 7	124.1	7.85^−29^
8. Two ratio (*ω*1, HDC; *ω*2, others)	*ω*1 = 0.4646; *ω*2 = 0.1638	−9,844.01	221	1 vs. 8	260.4	1.43^−58^
9. Two ratio (*ω*1, Yinpterochiroptera; *ω*2, Yangochiroptera	*ω*1 = 0.2448; *ω*2 = 0.1334	−9,855.28	221	1 vs. 9	282.9	1.75^−53^
10. Two ratio (*ω*1, echolocating; *ω*2, nonecholocating)	*ω*1 = 0.1836 ; *ω*2 = 0.2047	−9,868.35	221	1 vs. 10	309.1	3.50^−69^
11. Two ratio (*ω*1, primarily cave roosters; *ω*2, other)	*ω*1 = 0.2957; *ω*2 = 0.1219	−9,834.15	221	1 vs. 11	42.24	8.07^−11^
10. Two ratio (*ω*1, insectivore/carnivore; *ω*2, frugivore/nectivore)	*ω*1 = 0.1845 ; *ω*2 = 0.2014	−9,868.51	221	1 vs. 12	26.48	2.66^−7^
*SWS1* opsin gene: Pteropodidae						
1. One ratio	*ω* = 0.2247	−4,918.21	92			
2. Free ratio	Variable by branch	−4,823.60	181	1 vs. 2	189.2	6.50^−08^
3. M1a	*p*1 = 0.8135; *p*2 = 0.1865	−4,859.52	93			
	*ω*1 = 0.1086; *ω*2 = 1.0000					
4. M2a	*p*1 = 0.8135; *p*2 = 0.0489; *p*3 = 0.1376	−4,859.91	95	3 vs. 4	0.78	0.677
	*ω*1 = 0.1086; *ω*2 = 1; *ω*3 = 1					
5. M7β	*p* = 0.38351; *q* = 1.13584	−4,859.97	93			
6. M8 β&ω	*p*0 = 0.9499; *p*1 = 0.5836; *q* = 2.3549	−4,855.76	95	5 vs. 6	8.42	0.015
	(*p*2 = 0.0501); *ω* = 1.601					
7. Two ratio (*ω*1, nonfunctional; *ω*2, functional	*ω*1 = 0.5173; *ω*2 = 0.1154	−4,941.03	93	1 vs. 7	45.6	1.41^−11^
8. Two ratio (*ω*1, echolocating; *ω*2, nonecholocating)	*ω*1 = 0.6660; *ω*2 = 0.1684	−4,962.70	93	1 vs. 8	89	3.95^−21^
9. Two ratio (*ω*1, cave; *ω*2, tree)	*ω*1 = 0.5384; *ω*2 = 0.1132	−4,937.97	93	1 vs. 9	37.6	8.69^−10^

a The log likelihood values.

b Number of parameters.

c 2Δ = 2 × (ln *L*1 – ln *L*2).

Since the Pteropodidae rely mainly on vision rather than echolocation as their primary mode of sensory perception, we performed targeted selective pressure variation analyses within this group. The *ω* estimate across the Pteropodidae was 0.2247, confirming that the *SWS1* opsin gene is evolving under purifying selection within this bat family. Since the majority of cave roosting pteropodid bats have a nonfunctional *SWS1* opsin, the *ω* estimate for foreground branches “nonfunctional” and “cave roosting” were similar (*ω*_non__functional SWS1_ = 0.5173; *ω*_cave-roosting_ = 0.5384) and higher than their respective background comparative branches (*ω*_functional SWS1_ = 0.1154; *ω*_tree-roosting_ = 0.1132) (table 2). The site models M8 β&ω and M2a detected 7 and 4 sites under positive selection, respectively (table 2). These sites are located in transmembrane domains and extra and intra cellular loops.

### Dating the Loss of the *SWS1* Opsins

The sequencing and phylogenetic analysis of Cyt *b* completed gaps in the published phylogenies for Mormoopidae, Megadermatidae, and Pteropodidae (supplementary figs. S2–S4, Supplementary Material online). ML and BA estimated similar tree topologies, the majority of which were in agreement with published phylogenies, although some BS and BPP values were low (supplementary figs. S2–S4, Supplementary Material online). Within Mormoopidae, *Pteronotus personatus* was placed as basal to *Pteronotus quadridens*, *Pteronotus macleayii*, *Pteronotus gymnonotus*, and *Pteronotus davyi* (supplementary fig. S2, Supplementary Material online). In Megadermatidae, the *Cyt b* phylogeny did not support the monophyly of the genus *Megaderma* within the Megadermatidae (supplementary fig. S3, Supplementary Material online). Dating the divergence of the three families mentioned suggested that *Megaderma lyra* diverged from the other megadermatids around 26.6 Ma and *Macroderma gigas* diverged from *Lavia frons* 7.9 Ma (supplementary fig. S3, Supplementary Material online). Within Mormoopidae, *P. gymnonotus* diverged from *P. davyi* approximately 3.8 Ma. The genus *Mormoops* diverged from the other Mormoopidae 33.8 Ma and both *Mormoops* species diverged from each other approximately 17 Ma (supplementary fig. S2, Supplementary Material online). In the Pteropodidae, *Dyacopterus* diverged from *Saphaerias* 14.9 Ma, *Thoopterus* diverged 16.3 Ma from the other pteropodids, *Scotonycteris* diverged from *Casinycteris* 8.4 Ma, *Eonycteris* diverged approximately 17.8 Ma, and the crown-group *Rousettus* radiated 10.4 Ma (supplementary fig. S4, Supplementary Material online).

Divergence time estimates were consistent with finding from previous studies (supplementary table S1, Supplementary Material online) and indicated the oldest pseudogenization event in the *SWS1* opsin gene occurred in *Mormoops*, Rhinolophidae, and Hipposideridae approximately 29.8, 29.1, and 28.4 Ma, respectively. In the megadermatids, *Megaderma lyra* and *Macroderma gigas* lost the *SWS1* opsin approximately 4.06 and 2.86 Ma, respectively (supplementary figs. S2–S4, Supplementary Material online). Pseudogenization of the *SWS1* in the Pteropodidae occurred multiple times in the majority of the cave roosting bats ranging from 6.79 Ma in *Dobsonia* and 12.01 Ma in *Rousettus* (supplementary fig. S4, Supplementary Material online).

## Discussion

The functionality of the bat visual system has been debated for many years, with common misconceptions compounded in the popular simile “as blind as a bat.” However, genetic (Zhao, Rossiter, et al. 2009; Zhao, Ru, et al. 2009; Wu, Wang, and Hadly 2017), behavioral (Fujun et al. 2012), and immunocytochemical studies of the visual pigments (Müller and Peichl 2005; Müller, Goodman, and Peichl 2007) have revealed that earlier expectations of blindness or pure rod retinas in bats (Peichl 2005) were incorrect.

### *MWS/LWS* Evolution in Bats

Wang et al. (2004) proposed the *MWS/LWS* opsin gene had potentially undergone duplication in the pteropodid *Haplonycteris fisheri* and further suggested that the presence of two copies may increase the expression and sensitivity of this visual pigment in this species. Our sequence comparison of clones derived from each pteropodid species, examined as part of this study, did not recover any indels or mutations, indicative of additional copies of the *MWS/LWS* opsin gene (Yokoyama and Radlwimmer 1998; Wang et al. 2004). This result provides strong support for the presence of a single copy of the *MWS/LWS* opsin gene in the pteropodid species we examined, suggesting that any duplication event in the Pteropodidae is likely to be limited to the monotypic genus *Haplonycteris*. Together, these results suggest that the majority of bats are potentially dichromatic given the additional presence of a *SWS1* functional opsin. Our spectral tuning analysis of the five amino acid sites responsible for the *λ*_max_ revealed that the majority of bat *MWS/LWS* visual pigments are tuned to a long wavelength (∼555–560 nm) (fig. 1). Ancestral state reconstructions demonstrated that the ancestor of all bats and those of the four major bat lineages (Rhinolophoidea, Emballonuroidea, Noctilionoidea, and Vespertilionoidea) were tuned to 555 nm (fig. 1). An increase of *λ*_max_ to ∼560 nm was observed in the majority of species sampled from the families Vespertilionidae and Megadermatidae and also in isolated species within the Rhinolophidae, Hipposideridae, and Phyllostomidae (fig. 1). Given that the laurasiatherian ancestor had a red-tuned *MWS/LWS* opsin gene tuned to ∼555 nm (Yokoyama and Radlwimmer 1998), we show that the ancestral laurasiatherian pattern is retained in the majority of bat species (fig. 1).

Assays conducted in vitro have demonstrated that the light reflected by the tapetum lucidium in *Pteropus giganteus* is red tuned (Blackwood et al. 2010). This suggests that 555–560 nm wavelength sensitive vision is complemented by other visual abilities which may be particularly advantageous in nocturnal conditions. However, the *MWS/LWS* appears to have undergone a green-shift in two pteropodid species, *Dyacopterus rickarti* (*λ*_max_ ∼ 536 nm) and *Thoopterus nigrescens* (*λ*_max_ ∼ 536 nm), and one vespertilionid species, *Myotis ricketii* (*λ*_max_ ∼ 543 nm). *Myotis ricketii* exhibits the lowest frequency rhodopsin *λ*_max_ (497 nm) observed in Chiroptera (Levenson and Dizon 2003; Zhao, Ru, et al. 2009), suggesting that parallel *λ*_max_ changes in the *MWS/LWS* opsin gene may be adaptive. The medium-wavelength *MWS/LWS* shift observed in the two pteropodid species, *Dyacopterus rickarti* and *Thoopterus nigrescens*, may be linked with the loss of the *SWS1* opsin. Changes in spectral sensitivity in functional opsins as a consequence of the pseudogenization of the *SWS1* opsin has been suggested in cetaceans (Levenson and Dizon 2003; Meredith et al. 2013), prior to the discovery that the *MWS/LWS* opsin gene was nonfunctional in this lineage (Meredith et al. 2013). The *ω* estimates from our selection test analyses showed strong purifying selection acting on the *MWS/LWS* opsin gene in bats (table 1). This suggests that despite the acquisition of laryngeal echolocation and a long history of nocturnality, the *MWS/LWS* opsin gene has evolved under very strong functional constraint in bats since they diverged from other laurasiatherians (Cavallari et al. 2011; Meredith et al. 2011). The *MWS/LWS* opsin gene is however under significant lower functional constrain in HDC lineages (table 1), suggesting that this type of echolocation may be correlated with relaxed long-wavelength vision. This is also supported by codon-based likelihood clade models (Gutierrez et al. 2018). With the exception of cetaceans and fossorial rodents, the *MWS/LWS* opsin is highly conserved among mammals (Yokoyama and Radlwimmer 1998; Zhao, Rossiter, et al. 2009), which highlights the importance of medium to long wavelength vision for mammals. Therefore, *MWS/LWS* vision may be fundamental for bat survival and may play an important role in the regulation of circadian rhythms which calibrate physiological processes and behavior (Nowak 1994; Cavallari et al. 2011). Furthermore, the ability to process longer wavelengths may be more important for image formation than shorter wavelengths such as UV light (Douglas and Jeffery 2014). Longer wavelengths are used preferentially in luminance vision and achromatic intensity detection providing more signal power and thus visual information (Osorio and Vorobyev, 2005). The importance of long-wavelength vision is particularly evident since the *LWS* opsin in vertebrates originated from a gene duplication predating all other cone opsins (Yokoyama, 2000).

### *SWS1* Evolution in Bats: UV Vison, HDC Echolocation, and Cave Roosting

Previous studies have suggested that bats possess UV short-wavelength vision, based on analyses of the 11 amino acids responsible for the tuning of this visual pigment (Jacobs 2009; Zhao, Rossiter, et al. 2009). Although caution is recommended when predicting *λ*_max_ from *SWS1* amino acid sequences (Hauser, van Hazel, and Chang 2014), UV vision in bats is further supported by electroretinographic recording in two Phyllostomidae species, *Carollia perspicillata* and *Glossophaga soricina* (Wang et al. 2004; Müller et al. 2009) and behavioral studies (Fujun et al. 2012). Our spectral tuning analysis of the 11 sites responsible for light sensitivity in the *SWS1* opsin gene in both ancestral and extant bat species, provide further support for the presence of UV vision in bats. Furthermore, our results demonstrate that this visual pigment has been UV sensitive in all bats since they diverged from other laurasatherians ∼ 78 Ma (Foley, Springer, and Teeling 2016). UV-sensitive *SWS1* opsins have a slower retinal release, a smaller binding pocket, increased dark stability, and a narrower absorption curve (Hauser, van Hazel, and Chang 2014), which together suggest that this visual pigment is adapted to mesopic and scotopic (mid-dim light) conditions. Amongst mammals, a UV-sensitive *SWS1* opsin is associated with a nocturnal lifestyle (Veilleux and Cummings 2012) and is thought to be particularly advantageous at dawn and dusk (Peichl 2005; Müller et al. 2009; Zhao, Rossiter, et al. 2009; Zhao, Ru, et al. 2009; Jacobs 2013). This may be particularly important for nectar-feeding lineages such as the Phyllostomidae which use UV vision to detect UV-reflecting flowers (Winter and von Helversen 2003).

In mammals, the evolutionary history of the *SWS1* opsin gene is remarkably different compared with the highly conserved *MWS/LWS* or the *RH1* opsin (Zhao, Rossiter, et al. 2009; Zhao, Ru, et al. 2009). Our data show that the *SWS1* opsin gene has undergone multiple pseudogenization events in bats, evidenced by the presence of frame-shift indels and premature stop codons in key locations in many pteropodid and echolocating bat lineages (14 bat lineages in total, figs. 2 and 3). Previous studies have suggested a sensory trade-off between *SWS1* opsin functionality and HDC echolocation (Zhao, Rossiter, et al. 2009), however, for the first time, our analyses show that the *SWS1* opsin has undergone pseudogenization in more echolocating bat lineages and is not limited to HDC echolocators (fig. 2). All Old World HDC echolocators (families Rhinolophidae Hipposideridae, and Rhinonycteridae) showed loss-of-function mutations, however this was not observed in the only other bat species reported capable of HDC echolocation, the New World *Pteronotus parnellii*. This suggests that acquisition of HDC echolocation does not always coincide with loss of function of the *SWS1* opsin in bats, as previously suggested (Zhao, Rossiter, et al. 2009). Loss of functionality of the *SWS1* was also observed in low-duty cycle (LDC) echolocating species from the families Megadermatidae and Mormoopidae (figs. 2 and 3), suggesting a more complex interplay of echolocation and vision in bats. The presence of partial *SWS1* fragments in the *Mormoops blainvillei* retinal transcriptome suggest that the three deletions may result in a translated pseudogene (Gutierrez et al. 2018). Further pseudogenization in vampire bats (Kries et al. 2018) at similar time frames to other echolocating bat lineages (supplementary table S1, Supplementary Material online) suggests that loss of the *SWS1* opsin gene may be widespread across Chiroptera.

The loss of the *SWS1* opsin gene is observed in several pteropodid lineages due to presence of premature frame-shift indels and stop codons (figs. 2 and 3). These lineages include species which roost in caves (*Rousettus*, *Stenonycteris*, and *Eonycteris*), those which roost in caves and trees (*Eidolon*) and a subset with as yet uncharacterized roosting ecology (*Thoopterus* and *Dyacopterus*) (Nowak 1994). Preliminary observations suggest the genus *Dyacopterus* may have mixed roosting habits since it has been found roosting in trees but has also been caught near caves (Nowak 1994; Giannini and Simmons 2003). Previous hypotheses which suggest the pseudogenization of the *SWS1* opsin may be related to the adoption of cave roosting habits (Zhao, Rossiter, et al. 2009) is supported by the majority of the species sequenced as part of this study. However, two exceptions appear to contradict this pattern, specifically, the pseudogenization of the *SWS1* opsin in *Scotonycteris zenkeri* and the presence of a functional *SWS1* opsin in *Lissonycteris angolensis*, a cave roosting species. Potentially, these observed differences can be attributed to a very recent change in the roosting ecology of these species, which may have occurred so recently that this switch has not yet exerted an observable effect at the genomic level. Generally, it is accepted that the acquisition of cave roosting habits in many extant pteropodid genera may have occurred independently from an ancestral tree roosting habit (Giannini and Simmons 2003; Li et al. 2008). This fits well with our results and lends further support to the hypothesis that cave roosting ecology drives *SWS1* opsin loss in the Pteropodidae. The measurement of spectral reflectance of fruits and flowers under different twilight and full moon conditions for monochromat primates suggest that the loss of the *SWS1* may not be disadvantageous when the luminance contrasts of critical stimuli are high (Moritz, 2015).

### *SWS1* Loss and Ecological Niche Adaptation

The loss of gene function may hold no adaptive value, as in the case of hemoglobin in Antarctic icefish (Sidell and O’Brien 2006). It may also be a driver of novel phenotypes, as observed in rattlesnake venom phenotypes (Casewell, 2016). Results from this paper and previous studies show that the evolution of the *SWS1* opsin in mammals is radically different from the other cone visual pigments. Both the *SWS2* and *Rh2* cone opsins were lost in ancestor of eutherian mammals (Yokoyama and Shi 2000), most likely in response to inhabiting a nocturnal environment (Heesy and Hall 2010). In extant taxa, the occupation of extreme photic environments has led to the degeneration of the *SWS1* and the *LWS* opsin genes in some cetaceans (Mizutani et al. 2013; Springer et al. 2016) and in fossorial and subterranean rodents (Emerling and Springer 2014). Furthermore, in fossorial rodents the loss of visual pigments is associated with further inactivation of genes in the cone visual pathway (Emerling and Springer 2014). However, losses of function in the *SWS1* opsin gene have puzzled biologists for decades (reviewed in Jacobs et al. 2013) ever since they were first discovered in some nocturnal primates, nocturnal, aquatic carnivores, and in several rodent lineages (David-Gray et al. 2002; Levenson and Dizon 2003; Carvalho et al. 2006; Jacobs 2013). Interestingly, the *SWS1* appears to lose function over relatively short evolutionary time frames, as observed in the rodent family Sciuridae (Carvalho et al. 2006), in the bat families Pteropodidae, Mormoopidae, and Megadermatidae (fig. 2) and Lemoriforme primates (Tan et al. 2005; Jacobs 2013). Together these results suggest that the pseudogenization of the *SWS1* opsin may be a more widespread evolutionary event among mammals than was previously thought, the full extent of this event will be revealed through increased taxonomic sampling and sequencing.

The loss of functional *SWS1* opsins across mammals has been suggested to be nonadaptive, given that many of these pseudogenization events are not ecologically advantageous (Jacobs 2013). Previous studies have shown that among nocturnal mammals, the spectral tuning of the *SWS1* opsin appears to be strongly associated with ways of foraging, whereas the *MWS/LWS* opsin is tuned to maximize the amount of light that can be absorbed from a given environment (Veilleux and Cummings 2012). Significant shifts in selective pressures can be found in the bat *SWS1* in cave roosting species (table 2) and among vegetation foragers (Gutierrez et al. 2018) and lemurs that inhabit open canopy forests generally experience higher purifying selection in the *SWS1* when compared with closed canopy rainforests, which have lower short-wavelength light levels (Veilleux, Louis, and Bolnick 2013). These cases highlight the potential role of different ecological factors in *SWS1* evolution. A loss of function in *SWS1* has also been hypothesized as having an adaptive role in species where luminance contrasts for critical stimuli, specifically gums/saps and flowers are high (Moritz, 2015), and may explain its rapid loss of function in the frugivourous Pteropodidae. The short time frame in which *Pteronotus parnellii* has developed HDC echolocation poses important questions: How fast can HDC echolocation evolve or have other mormoopids regressed to a LDC echolocation during their evolutionary histories? Is it possible that HDC echolocation was more widespread over the course of bat evolutionary history? To date, it is not known how and why HDC echolocation arose in bats or if any of the extant LDC lineages used HDC echolocation at some point in their evolutionary history (Fenton, Faure, and Ratcliffe 2012). Although Doppler shift compensation is mainly used by HDC bats, it also used by LDC bats, such as *Pteronotus personatus*, which demonstrates that LDC bats have the potential to develop their call emission and information processing capabilities (Smotherman and Guillén-Servent 2008). The observed convergent phylogenetic signal observed in the *SWS1* opsin gene (supplementary fig. S1, Supplementary Material online) suggests that echolocation may be linked to the evolution of photoreception in bats, possibly as consequence of changes in habitat and their visual system.

## Conclusion

Our data suggest the pseudogenization of the *SWS1* opsin gene is more widespread in bats than was previously thought. Our data confirm the loss of short-wavelength vision in the Old World HDC echolocators (Rhinolophidae, Hipposideridae, and Rhinonycteridae); conversely our data show that the New World HDC echolocator *Pteronotus parnellii* has a functional *SWS1*. The evolution of HDC echolocation is a relatively recent event in *Pteronotus parnellii* compared with the Old World HDC echolocators. This suggests that the short period of time between the divergence of *Pteronotus parnellii* and other *Pteronotus* species may not have been sufficient to relax the functionality of the *SWS1* opsin gene. The adoption of cave roosting habits is highly correlated with pseudogenization of the *SWS1* opsin gene in the Pteropodidae. Although, typically the loss of *SWS1* in mammals appears to be nonadaptive, potentially bats may have evolved mechanisms to compensate for the loss of UV vision. This study highlights the utility of studying sensory ecology and adaptation across divergent taxa to elucidate the diversity of selective pressures driving the evolution of vision in mammals.

## Materials and Methods

### Taxon and Genic Coverage

The wide sensory and ecological diversity represented in extant bat taxa is reflected in the taxonomic representation of all data sets included in this study. The *SWS1* opsin gene (exons 1–4) was amplified, cloned and sequenced (∼2.2–2.8 kb) in echolocating bat species across the suborders Yangochiroptera and Yinpterochioptera and included representatives from all but one (Cistugidae) currently recognized bat families (Teeling, Jones, and Rossiter 2016) (supplementary tables S2 and S3, Supplementary Material online). Exons 1–5 were amplified in a subset of species, including pteropodid species, to confirm the presence of a full-length open reading frame (ORF) in these taxa (supplementary table S2, Supplementary Material online). In addition to the *SWS1* gene, the *MWS/LWS* opsin gene (exons 3–5) was amplified, cloned, and sequenced (∼3.5 kb) in 18 echolocating and 7 nonecholocating (Pteropodidae) bat species (supplementary tables S2 and S3, Supplementary Material online). To ensure our results were robust we sequenced multiple (1–5) specimens per species. Both novel data sets were supplemented with data from previous analyses (Wang et al. 2004; Zhao, Rossiter, et al. 2009; Shen et al. 2010) and with data mined from the publicly available *Myotis lucifugus* and *Pteropus vampyrus* genomes (Ensembl v63) and consisted of 115 bat species for the *SWS1* opsin gene and 45 species for *MWS/LWS* opsin gene. GenBank accession numbers and detailed sample information are provided in supplementary table S2, Supplementary Material online. A total of 11 mammals from the laurasiatherian orders Carnivora, Cetartiodactyla, Perissodactyla, and Eulipotyphla were chosen as outgroups for downstream phylogenetic analyses (supplementary table S2, Supplementary Material online).

For a subset of samples, species identity was confirmed through either *CO1* or Cyt *b* sequencing. A total of 44 samples were barcoded through the amplification and sequencing of the *CO1* gene. Given that certain analyses, such as selection analyses in PAML, require a resolved bifurcating phylogeny, Cyt *b* was amplified and sequenced for six samples to ascertain their phylogenetic positions within Mormoopidae and Megadermatidae (see below) (supplementary table S2, Supplementary Material online).

### DNA Alignments

DNA sequences were aligned with a combination of MAFFT v6 (Katoh et al. 2002), ClustalX (Larkin et al. 2007) and Muscle (Edgar 2004) and manually refined in Geneious (Kearse et al. 2012). Intron/Exon boundaries were identified by the GT/AG boundary rule and by aligning them with published mRNA sequences, following Zhao, Rossiter, et al. (2009). Exons with variable length compared with reference sequences were considered splice variants. Introns were identified through comparison to reference sequences and subsequently removed to create alignments of opsin coding/exonic regions. Sequences which contained premature stop codons or frame-shift indels were designated as nonfunctional. A total of four alignments (3 *SWS1* and 1 *MWS/LWS*) were generated. As exon 5 was sequenced in only a subset of taxa, the first alignment consisted of *SWS1* exons 1–5, with the fifth exon encoded as missing data in relevant taxa. The second alignment consisted of the full *SWS1* opsin exons 1–4 only, for all taxa. The final *SWS1* alignment consisted of functional opsin genes only (exon 1–5). The *MWS/LWS* alignment contained opsin genes for all taxa sequenced.

### Phylogenetic Analyses

jModelTest v3.7 (Posada 2008) was used to find the best fitting model of sequence evolution for each gene fragment. GTR+I + G was the best fitting model for all *SWS1* opsin data sets. HKY+G was the best fit model for *MWS/LWS* opsin gene. All model fits were assessed using the AIC.

RAxML v7.2 (Stamatakis 2006) and Mr.Bayes v3.1.2 (Ronquist and Huelsenbeck 2003) were used generate ML and BA gene trees for each gene. ML analyses were executed in RAxML using majority rule (MRE)-based bootstrapping criteria; randomized MP starting trees, a fast hill-climbing algorithm, and the best fit model of sequence evolution. The BA included 1,100,000 generations with chains sampled every 1,000 generations, random starting trees, 4 chains (3 hot and 1 cold). Convergence was assessed through monitoring of the standard deviation of split frequencies and stationarity was reached when the standard deviation of split frequencies fell below 0.01.

### dN/dS Estimates

The CodeML package, implemented in PAML 4.4 (Yang 2007), was used to estimate the ratio of the rates of nonsynonymous substitutions (dN) to synonymous substitutions (dS; dN/dS or *ω*) for the *SWS1* (exon 1–4) data set for all bats, *SWS1* in Pteropodidae (exon 1–4) and the *MWS/LWS* opsin gene. Given that dN/dS analyses do not allow the presence of indels, codons containing indels were removed from all sequences prior to analysis. The composite species tree used in the CodeML analysis was based on chiropteran intraordinal relationships from Teeling et al. (2005), the Mormoopidae topology from Dávalos (2006), the Phyllostomidae topology from Rojas et al. (2011), the Rhinolophidae topology from Stoffberg et al. (2010), the Hipposideridae topology from Sun et al. (2009), and the Pteropodidae phylogeny from Almeida et al. (2011) (supplementary figs. S5 and S6, Supplementary Material online). PAML analyses were used on alignments of both functional *SWS1* sequences only and alignments where stop codons/indels in species were removed from the sequence.

Branch models allow the *ω* ratio to vary in branches across a given topology and therefore can detect positive selection, where *ω*  >  1, in lineages of interest. The simplest branch model (one ratio) only allows one *ω* ratio across the tree, whereas the more complex free ratio assumes independent *ω* ratios for each branch. Branch models for the *SWS1* in all bats and *MWS/LWS* opsin genes were used to estimate the *ω* ratio for four foreground lineages: HDC echolocating bats, nonecholocating bats (Pteropodidae), primarily cave roosting bats, insectivore/carnivore bats, and lineages with a nonfunctional *SWS1* opsin gene. In the Pteropodidae *SWS1* data set, branch models were performed on the foreground lineages of cave roosting pteropodids, *SWS1* nonfunctional pteropodids and the tongue-click echolocators. All branch models were compared with the simplest models (one ratio) using a likelihood ratio test (LRT) and accepted if *p* > 0.05. Site models (M1a nearly neutral, M2a positive selection and M7β, M8 β&*ω*) allow *ω* ratios to vary among sites (amino acid or codons). Site models M2a and M8 β&ω were compared by LRT with site models M1a and M7β, respectively. Bayes empirical Bayes (BEB) (Yang, Wong, and Nielsen 2005) implemented in models M2a and M8 β&*ω* was used to identify sites evolving under positive selection across the opsin genes.

HyPhy (Pond, Frost, and Muse 2005) was used to identify directional episodic selection in the *SWS1* and *MWS/LWS* opsin genes across branches in bats (BranchSiteREL) (Kosakovsky et al. 2011). A mixed effects model of episodic (MEME) selection (Murrell et al. 2012) and fast unconstrained Bayesian approximation (FUBAR) (Murrell et al. 2013) was used to detect episodic selection on amino acid sites and was implemented in Datamonkey (Delport et al. 2010).

### Ancestral State Reconstruction

Ancestral sequences were inferred using a combination of Bayesian and Parsimony methods using PAML and Ancestors v1.1 (Yang 2007; Diallo, Makarenkov, and Blanchette 2010). Given that the Bayesian method (Yang 2007) implemented in PAML does not accept indels, it was used to infer the ancestral sequence in alignments that excluded indels. Ancestors v1.1, which uses heuristic and tree-hidden Markov models, was used to reconstruct ancestral regions containing indels in the full-length *SWS1* alignment. Ancestral sequences were aligned with extant opsin data and spectral sites were identified. Despite possible allelic variations to the “five-site” rule observed in certain vertebrate taxa, ancestral wavelength of peak sensitivity (*λ*_max_) was reconstructed using inferred *λ*_max_ values based on amino acid composition of the five key sites observed across each bat species (Yokoyama, Yang, and Starmer 2008).

### Dating Loss of Function in *SWS1*

Divergence time estimates were determined to date pseudogenization events in key lineages. Although, the timing of the basal divergence within the Megadermatidae, Pteropodidae, and Mormoopidae has been estimated by previous studies (Teeling et al. 2005; Miller-Butterworth et al. 2007; Meredith et al. 2011; Foley et al. 2015), the phylogenetic relationships and timing of diversification events within these families have not yet been resolved. Publicly available Cyt *b* sequence data from representatives of the Megadermatidae, Pteropodidae, and Mormoopidae were downloaded and gaps in taxonomic representation were assessed. New sequence data was generated for key species which were not present in the publicly available Cyt *b* data set using methods as described above. Novel Cyt *b* data was aligned with publicly downloaded sequences using MAFFT v6 (Katoh et al. 2002). jModelTest 3.7 (Posada 2008) was used to determine the best fitting model of sequence evolution for the data, according to the AIC or BIC scores. ML analysis was performed as previously described with the GTR model for the Pteropodidae data set (outgroup Rhinolophoidea) and GTR+I + G for the Mormoopidae data set (outgroup: Phyllostomidae) and Rhinolophoidea data set (outgroup: Pteropodidae). Phylogenetic reconstruction and divergence time estimates for these data sets were estimated simultaneously using BEAST v1.7 (Drummond and Rambaut 2007). The uncorrelated, lognormal, relaxed-clock model, where rates were allowed to vary among branches without the *a priori* assumption of autocorrelation between adjacent branches, was used. Default priors were used for GTR substitution parameters (0, 100), gamma shape parameter (0, 100), and proportion of invariant sites parameter (0, 1). The uncorrelated lognormal clock was estimated with uniform priors on the mean (0, 100) and standard deviation (0, 10). The Yule process of speciation was used as the tree prior and the starting tree was estimated with UPGMA. The ingroup was assumed to be monophyletic with respect to the outgroup. Phylogenetic calibrations were applied to several branches of the tree to constrain the analysis. (1) the split between the Pteropodidae and all other yinpterochiropterans was constrained following hard bound maximum age estimates (63.79 My) obtained in Meredith et al. (2011) and (2) the crown (24 My) group Pteropodidae was constrained following estimates obtained in the analysis of Teeling et al. (2005). The calibration point for the Megadermatidae was based on the divergence times of the split between *Craseonycteris + Megaderma* (44 My) estimated in the study of Foley et al. (2015). For Mormoopidae, the calibration point was based on the hard-bound maximum age divergence time estimate for the split between Phyllostomidae + Mormoopidae (37.11 My), from Meredith et al. (2011).

For each data set, three independent Markov chain Monte Carlo (MCMC) were run for 30 million generations to ensure sufficient sampling of estimated sample size (ESS) values, with auto optimize operators. Trees were saved every 1,000 generations. Log files from each run were imported into Tracer v1.7 (Rambaut et al. 2007), with trees sampled from the first 1 million generations discarded as burn-in and stationarity was assessed. Tree files from the individual runs were combined using LogCombiner v1.7 (Drummond and Rambaut 2007) to find the optimal tree.

### Dating Pseudogenization Events and Estimating Ecological Correlations

In lineages where the *SWS1* gene is pseudogenized (figs. 2 and 3), we estimated when functional and evolutionary constraints were relaxed using the method described in Zhao et al. (2010). This method assumes a scenario in which the typical evolutionary constraint (as observed in other closely related bat lineages) was suddenly and completely relaxed (*ω* = 1) at some time point in evolutionary history (*t* My), as such our analyses used: (1) the rate of nucleotide substitution increase in lineages with a pseudogenized *SWS1* gene and (2) the *ω* value prior to the relaxation of the functional constraint in closely related functional lineages. The data set was subdivided into four subsets to limit the exclusion of codons due to the presence of indels in the alignments: (1) *Pteronotus*; (2) Mormoopidae + Phyllostomidae; (3) Rhinolophoidea; and (4) Pteropodidae. For each of these data sets we used the formula detailed in Zhao et al. (2010) based on functional and nonfunctional lineage *ω* estimates, assuming divergence estimates obtained as part of this study.

Additionally, the method developed by Meredith et al. (2009), in which CodeML (Yang 2007) was used to estimate the dN/dS ratios or *ω* in four branch categories: functional (pre-existing to functional branches); premutation (predating internal nodes where stop codons appear); mixed (presence of first-appearance stop codons and anteceding nonfunctional branches), and nonfunctional (postdate first-appearance stop codons). This analysis was applied to three data sets: (1) Mormoopidae; (2) Rhinolophoidea, and (3) Pteropodidae. Subsequently, we used *ω* estimates from each branch category to resolve the mixed branches into their functional and pseudogenic components. The times of divergence for each lineage were estimated in the previous section.

To estimate possible associations between the loss of *SWS1* opsin genes and the acquisition of primarily cave roosting habitats in Pteropodidae, the Brunch algorithm in Caper (Orme 2012), as implemented in R (R Development Core Team 2013), was used with a pteropodid species tree. However, there currently exists no accurate models to estimate nodal values for categorical variables (Orme 2012).

### Estimates of Convergent Evolution

Alternative topology tests were used to assess the relative support for the convergent gene tree topology versus the species tree topology (where Yinpterochiroptera was constrained) using the functional *SWS1* data set. Site-wise likelihood values were then used to conduct an approximately unbiased (AU) test (Shimodaira 2002) in Consel (Shimodaira and Hasegawa 2001). Methods described by Liu et al. (2011) and Castoe et al. (2009) were used to identify convergent amino acids in the echolocating bat species. Amino acid changes per branch were estimated based on the marginal ancestral sequence reconstructions generated by PAML through comparison of states at ancestral and descendant nodes in the Rhinolophoidea and Yangochiroptera. For amino acid sites at which changes occurred along the two compared branches, sites with different amino acids in the descendants were defined as divergent, and those with the same amino acid in the descendant were defined as convergent.

## Supplementary Material

Supplementary data are available at *Molecular Biology and Evolution* online.

## Supplementary Material

Supplementary Data

## References

[msy192-B1] Almeida FC, Giannini NP, DeSalle R, Simmons NB. 2011 Evolutionary relationships of the old world fruit bats (Chiroptera, Pteropodidae): Another star phylogeny? BMC Evol Biol. 11:281. 21961908 10.1186/1471-2148-11-281 PMC3199269

[msy192-B2] Blackwood SE, Plummer CE, Crumley W, MacKay EO, Brooks DE, Barrie KP. 2010 Ocular parameters in a captive colony of fruit bats. Vet Ophthalmol. 13:72–79. 20840093 10.1111/j.1463-5224.2010.00816.x

[msy192-B4] Bowmaker JK, Hunt DM. 2006 Evolution of vertebrate visual pigments. Curr Biol. 16(13):R484–R489. 16824904 10.1016/j.cub.2006.06.016

[msy192-B5] Carvalho LS, Cowing JA, Wilkie SE, Bowmaker JK, Hunt DM. 2006 Shortwave visual sensitivity in tree and flying squirrels reflects changes in lifestyle. Curr Biol. 16(3):R81–R83. 16461266 10.1016/j.cub.2006.01.045

[msy192-B6] Casewell NR. 2016 Venom evolution: Gene loss shapes phenotypic adaptation. Curr Biol. 26:R838–R858. 27676304 10.1016/j.cub.2016.07.082

[msy192-B165] Castoe TA, de Koning APJ, Kim H-M, Gu W, Noonan BP, Naylor G, Jiang ZJ, Parkinson CL, Pollock DD. 2009 Evidence for an ancient adaptive episode of convergent molecular evolution. Proc Nat Acad Sci USA 106:8986–8991. 19416880 10.1073/pnas.0900233106 PMC2690048

[msy192-B7] Cavallari N, Frigato E, Vallone D, Fröhlich N, Lopez-Olmeda JF, Foà A, Berti R, Sánchez-Vázquez FJ, Bertolucci C, Foulkes NS. 2011 A blind circadian clock in cavefish reveals that opsins mediate peripheral clock photoreception. PLoS Biol. 9(9):e1001142. 21909239 10.1371/journal.pbio.1001142 PMC3167789

[msy192-B8] Dávalos L. 2006 The geography of diversification in the mormoopids (Chiroptera: Mormoopidae). Biol J Linn Soc 88:101–118.

[msy192-B9] David-Gray Z, Bellingham J, Munoz M, Avivi A, Nevo E, Foster R. 2002 Adaptive loss of ultraviolet‐sensitive/violet‐sensitive (UVS/VS) cone opsin in the blind mole rat (*Spalax ehrenbergi*). Eur J Neurosci. 16(7):1186–1194. 12405979 10.1046/j.1460-9568.2002.02161.x

[msy192-B10] Davies KT, Bennett NC, Tsagkogeorga G, Rossiter SJ, Faulkes CG. 2015 Family wide molecular adaptations to underground life in African mole-rats revealed by phylogenomic analysis. Mol Biol Evol. 32(12):3089–3107. 26318402 10.1093/molbev/msv175 PMC4652621

[msy192-B11] Delport W, Poon AF, Frost SD, Kosakovsky Pond SL. 2010 Datamonkey 2010: A suite of phylogenetic analysis tools for evolutionary biology. Bioinformatics 26(19):2455–2457. 20671151 10.1093/bioinformatics/btq429 PMC2944195

[msy192-B12] Diallo AB, Makarenkov V, Blanchette M. 2010 Ancestors 1.0: A web server for ancestral sequence reconstruction. Bioinformatics 26(1):130–131. 19850756 10.1093/bioinformatics/btp600

[msy192-B13] Douglas R, Jeffery G. 2014 The spectral transmission of ocular media suggests ultraviolet sensitivity is widespread among mammals. Proc R Soc B. 281(1780):20132995. 10.1098/rspb.2013.2995 PMC4027392 24552839

[msy192-B14] Drummond AJ, Rambaut A. 2007 BEAST: Bayesian evolutionary analysis by sampling trees. BMC Evol. Biol. 7:214. 17996036 10.1186/1471-2148-7-214 PMC2247476

[msy192-B15] Edgar RC. 2004 MUSCLE: Multiple sequence alignment with high accuracy and high throughput. Nucleic Acids Res. 32(5):1792–1797. 15034147 10.1093/nar/gkh340 PMC390337

[msy192-B16] Eklöf J, Šuba J, Petersons G, Rydell J. 2014 Visual acuity and eye size in five European bat species in relation to foraging and migration strategies. Environ Exp Biol. 12:1–6.

[msy192-B17] Emerling CA, Huynh HT, Nguyen MA, Meredith RW, Springer MS. 2015 Spectral shifts of mammalian ultraviolet-sensitive pigments (short wavelength-sensitive opsin 1) are associated with eye length and photic niche evolution. Proc R Soc B. 282(1819):20151817. 10.1098/rspb.2015.1817 PMC4685808 26582021

[msy192-B18] Emerling CA, Springer MS. 2014 Eyes underground: Regression of visual protein networks in subterranean mammals. Mol Phylogenet Evol. 78:260–270. 24859681 10.1016/j.ympev.2014.05.016

[msy192-B19] Emerling CA, Springer MS. 2015 Genomic evidence for rod monochromacy in sloths and armadillos suggests early subterranean history for Xenarthra. Proc R Soc B 282(1800):20142192. 10.1098/rspb.2014.2192 PMC4298209 25540280

[msy192-B20] Fenton MB, Faure PA, Ratcliffe JM. 2012 Evolution of high duty cycle echolocation in bats. J Exp Biol. 215(Pt 17):2935–2944. 22875762 10.1242/jeb.073171

[msy192-B21] Foley NM, Springer MS, Teeling EC. 2016 Mammal madness: Is the mammal tree of life not yet resolved? Philos Trans Royal Soc B. 371(1699):20150140. 10.1098/rstb.2015.0140 PMC4920340 27325836

[msy192-B22] Foley NM, Thong VD, Soisook P, Goodman SM, Armstrong KN, Jacobs DS, Puechmaille SJ, Teeling EC. 2015 How and why overcome the impediments to resolution: Lessons from rhinolophid and hipposiderid bats. Mol Biol Evol. 32(2):313–333. 25433366 10.1093/molbev/msu329 PMC4769323

[msy192-B23] Fujun X, Kailiang H, Tengteng Z, Paul R, Xuzhong W, Yi S. 2012 Behavioral evidence for cone-based ultraviolet vision in divergent bat species and implications for its evolution. Zoologia 29:109–114. 10.1016/j.cbpb.2012.01.005 22269122

[msy192-B24] Giannini NP, Simmons NB. 2003 A phylogeny of megachiropteran bats (Mammalia: Chiroptera: Pteropodidae) based on direct optimization analysis of one nuclear and four mitochondrial genes. Cladistics 19(6):496–511. 10.1111/j.1096-0031.2003.tb00385.x 34905855

[msy192-B25] Griebel U, Schmid A. 1992 Color vision in the California sea lion (*Zalophus californianus*). Vision Res. 32(3):477–482. 1604834 10.1016/0042-6989(92)90239-f

[msy192-B26] Gutierrez EA, Schott RK, Preston MW, Loureiro LO, Lim BK, Chang BS. 2018 The role of ecological factors in shaping bat cone opsin evolution. Proc R Soc B. 285(1876):20172835. 10.1098/rspb.2017.2835 PMC5904313 29618549

[msy192-B27] Hauser FE, van Hazel I, Chang BS. 2014 Spectral tuning in vertebrate short wavelength‐sensitive 1 (SWS1) visual pigments: Can wavelength sensitivity be inferred from sequence data? J Exp Zool B Mol Dev Evol. 322(7):529–539. 24890094 10.1002/jez.b.22576

[msy192-B28] Heesy CP, Hall MI. 2010 The nocturnal bottleneck and the evolution of mammalian vision. Brain Behav Evol. 75(3):195–203. 20733295 10.1159/000314278

[msy192-B29] Hunt DM, Dulai KS, Cowing JA, Julliot C, Mollon JD, Bowmaker JK, Li W-H, Hewett-Emmett D. 1998 Molecular evolution of trichromacy in primates. Vision Res. 38(21):3299–3306. 9893841 10.1016/s0042-6989(97)00443-4

[msy192-B32] Jacobs GH. 2009 Evolution of colour vision in mammals. Philos Trans Royal Soc B. 364(1531):2957–2967. 10.1098/rstb.2009.0039 PMC2781854 19720656

[msy192-B33] Jacobs GH. 2013 Losses of functional opsin genes, short-wavelength cone photopigments, and color vision — a significant trend in the evolution of mammalian vision. Vis. Neurosci. 30:39–53. 23286388 10.1017/S0952523812000429

[msy192-B34] Katoh K, Misawa K, Kuma K-i, Miyata T. 2002 MAFFT: A novel method for rapid multiple sequence alignment based on fast Fourier transform. Nucleic Acids Res. 30(14):3059–3066. 12136088 10.1093/nar/gkf436 PMC135756

[msy192-B35] Kawamura S, Kasagi S, Kasai D, Tezuka A, Shoji A, Takahashi A, Imai H, Kawata M. 2016 Spectral sensitivity of guppy visual pigments reconstituted in vitro to resolve association of opsins with cone cell types. Vision Res. 127:67–73. 27476645 10.1016/j.visres.2016.06.013

[msy192-B36] Kearse M, Moir R, Wilson A, Stones-Havas S, Cheung M, Sturrock S, Buxton S, Cooper A, Markowitz S, Duran C, et al 2012 Geneious Basic: An integrated and extendable desktop software platform for the organization and analysis of sequence data. Bioinformatics 28(12):1647–1649. 22543367 10.1093/bioinformatics/bts199 PMC3371832

[msy192-B37] Kim EB, Fang X, Fushan AA, Huang Z, Lobanov AV, Han L, Marino SM, Sun X, Turanov AA, Yang P, et al 2011 Genome sequencing reveals insights into physiology and longevity of the naked mole rat. Nature 479(7372):223–227. 21993625 10.1038/nature10533 PMC3319411

[msy192-B38] Kosakovsky Pond SL, Murrell B, Fourment M, Frost SD, Delport W, Scheffler K. 2011 A random effects branch-site model for detecting episodic diversifying selection. Mol Biol Evol. 28(11):3033–3043. 21670087 10.1093/molbev/msr125 PMC3247808

[msy192-B172] Kos M., Bulog B, Szel A., Rohlich P. 2001 Immunocytochemical demonstration of visual pigments in the degenerate retinal and pineal photoreceptors of the blind cave salamander (*Proteus anguinus*). Cell Tissue Res. 303(1):15–25. 11236001 10.1007/s004410000298

[msy192-B39] Kries K, Barros MAS, Duytschaever G, Orkin JD, Janiak MC, Pessoa DMA, Melin AD. 2018 Colour vision variation in leaf-nosed bats (Phyllostomidae): Links to cave roosting and dietary specialization. Mol. Ecol. 18:3627–3640. 10.1111/mec.14818 30059176

[msy192-B40] Larkin MA, Blackshields G, Brown NP, Chenna R, McGettigan PA, McWilliam H, Valentin F, Wallace IM, Wilm A, Lopez R, et al 2007 Clustal W and Clustal X version 2.0. Bioinformatics 23(21):2947–2948. 17846036 10.1093/bioinformatics/btm404

[msy192-B41] Levenson DH, Dizon A. 2003 Genetic evidence for the ancestral loss of short-wavelength-sensitive cone pigments in mysticete and odontocete cetaceans. Proc R Soc B. 270(1516):673–679. 10.1098/rspb.2002.2278 PMC1691291 12713740

[msy192-B42] Li G, Wang J, Rossiter SJ, Jones G, Cotton JA, Zhang S. 2008 The hearing gene Prestin reunites echolocating bats. Proc Natl Acad Sci U S A. 105(37):13959–13964. 18776049 10.1073/pnas.0802097105 PMC2544561

[msy192-B43] Liu Y, Chi H, Li L, Rossiter SJ, Zhang S. 2018 Molecular data support an early shift to an intermediate-light niche in the evolution of mammals. Mol Biol Evol. 12:1131. 10.1093/molbev/msy019 29462332

[msy192-B162] Liu Z., Li S, Wang W, Xu D, Murphy RW, Shi P. 2011 Parallel evolution of KCNQ4 in echolocating bats. PLoS ONE 6(10):e26618. 22046315 10.1371/journal.pone.0026618 PMC3200345

[msy192-B44] Matsumoto Y, Hiramatsu C, Matsushita Y, Ozawa N, Ashino R, Nakata M, Kasagi S, Di Fiore A, Schaffner CM, Aureli F, et al 2014 Evolutionary renovation of L/M opsin polymprhism confers a fruit discrimination advantage to ateline New World monkeys. Mol Ecol. 23(7):1799–1812. 24612406 10.1111/mec.12703 PMC4260670

[msy192-B158] Meredith RW, Gatesy J, Murphy WJ, Ryder OA, Springer MS. 2009 Molecular decay of the tooth gene enamelin (ENAM) mirrors the loss of enamel in the fossil record of placental mammals. PLoS Genet 5:e1000634. 19730686 10.1371/journal.pgen.1000634 PMC2728479

[msy192-B45] Meredith RW, Gatesy J, Emerling CA, York VM, Springer MS. 2013 Rod monochromacy and the coevolution of cetacean retinal opsins. PLoS Genet. 9(4):e1003432. 23637615 10.1371/journal.pgen.1003432 PMC3630094

[msy192-B46] Meredith RW, Janečka JE, Gatesy J, Ryder OA, Fisher CA, Teeling EC, Goodbla A, Eizirik E, Simão TLL, Stadler T, et al 2011 Impacts of the cretaceous terrestrial revolution and KPg extinction on mammal diversification. Science 334(6055):521–524. 21940861 10.1126/science.1211028

[msy192-B47] Miller-Butterworth CM, Murphy WJ, O’Brien SJ, Jacobs DS, Springer MS, Teeling EC. 2007 A family matter: Conclusive resolution of the taxonomic position of the long-fingered bats, *Miniopterus*. Mol Biol Evol. 24(7):1553–1561. 17449895 10.1093/molbev/msm076

[msy192-B48] Mizutani Y, Tomita N, Niizuma Y, Yoda K. 2013 Environmental perturbations influence telomere dynamics in long-lived birds in their natural habitat. Biol Lett. 9(5):20130511. 23945210 10.1098/rsbl.2013.0511 PMC3971690

[msy192-B49] Mohun S, Davies W, Bowmaker J, Pisani D, Himstedt W, Gower D, Hunt D, Wilkinson M. 2010 Identification and characterization of visual pigments in caecilians (Amphibia: Gymnophiona), an order of limbless vertebrates with rudimentary eyes. J Exp Biol. 213(20):3586–3592. 20889838 10.1242/jeb.045914

[msy192-B50] Moritz GL. 2015 Primate Origins through the Lens of Functional and Degenerate Opsins. Hanover, New Hampshire. Dartmouth College. pp. 1–176.

[msy192-B51] Müller B, Glösmann M, Peichl L, Knop GC, Hagemann C, Ammermüller J. 2009 Bat eyes have ultraviolet-sensitive cone photoreceptors. PLoS One 4(7):e6390. 19636375 10.1371/journal.pone.0006390 PMC2712075

[msy192-B52] Müller B, Goodman SM, Peichl L. 2007 Cone photoreceptor diversity in the retinas of fruit bats (Megachiroptera). Brain Behav Evol. 70(2):90–104. 17522478 10.1159/000102971

[msy192-B53] Müller B, Peichl L. 2005 Retinal cone photoreceptors in microchiropteran bats. Invest Ophthalmol Vis Sci. 46:2259–2259.

[msy192-B54] Muller B, Peichl L, Winter Y, Von Helversen O, Glösmann M. 2007 Cone photoreceptors and ultraviolet vision in the flower bat Glossophaga soricina (Microchiroptera, Phyllostomidae). Invest Ophthalmol Vis Sci. 48:5951–5951.

[msy192-B55] Murrell B, Moola S, Mabona A, Weighill T, Sheward D, Kosakovsky Pond SL, Scheffler K. 2013 FUBAR: A fast, unconstrained bayesian approximation for inferring selection. Mol Biol Evol. 30(5):1196–1205. 23420840 10.1093/molbev/mst030 PMC3670733

[msy192-B56] Murrell B, Wertheim JO, Moola S, Weighill T, Scheffler K, Pond SLK. 2012 Detecting individual sites subject to episodic diversifying selection. PLoS Genet. 8(7):e1002764. 22807683 10.1371/journal.pgen.1002764 PMC3395634

[msy192-B152] Nathans J. 1990 Determinants of visual pigment absorbance: role of charged amino acids in the putative transmembrane segments. Biochemistry 29:937. 2111169 10.1021/bi00456a013

[msy192-B57] Nowak RM. 1994 Walker’s bats of the world. Baltimore and London: John Hopkins University Press.

[msy192-B58] Orme D. 2012 The caper package: Comparative analysis of phylogenetics and evolution in R. R Project Publication, http://CRAN.R-project.org/package=caper; accessed May 2018.

[msy192-B59] Osorio D, Vorobyev M. 2005 Photoreceptor spectral sensitivities in terrestrial animals: Adaptations for luminance and colour vision. Proc R Soc B. 272 (1574):1745–1752. 10.1098/rspb.2005.3156 PMC1559864 16096084

[msy192-B60] Peichl L. 2005 Diversity of mammalian photoreceptor properties: Adaptations to habitat and lifestyle? Anat Rec. 287(1):1001–1012. 10.1002/ar.a.20262 16200646

[msy192-B61] Pond SLK, Frost SD, Muse SV. 2005 HyPhy: Hypothesis testing using phylogenies. Bioinformatics 21(5):676–679. 15509596 10.1093/bioinformatics/bti079

[msy192-B62] Posada D. 2008 jModelTest: Phylogenetic model averaging. Mol Biol Evol. 25(7):1253–1256. 18397919 10.1093/molbev/msn083

[msy192-B63] R Development Core Team. 2013 R: A language and environment for statistical computing. ed. R Foundation for Statistical Computing, Vienna, Austria. http://www.R-project.org/; accessed May 2018.

[msy192-B64] Rambaut A, Suchard MA, Xie D, Drummond AJ. (2007) Tracer v1.4. http://tree.bio.ed.ac.uk/software/tracer/; accessed September 2011.

[msy192-B65] Rojas D, Vale A, Ferrero V, Navarro L. 2011 When did plants become important to leaf-nosed bats? Diversification of feeding habits in the family Phyllostomidae. Mol Ecol. 20(10):2217–2228. 21481051 10.1111/j.1365-294X.2011.05082.x

[msy192-B66] Ronquist F, Huelsenbeck JP. 2003 MrBayes 3: Bayesian phylogenetic inference under mixed models. Bioinformatics 19(12):1572–1574. 12912839 10.1093/bioinformatics/btg180

[msy192-B67] Schmidt S, Yapa W, Grunwald J-E. 2011 Echolocation behaviour of *Megaderma lyra* during typical orientation situations and while hunting aerial prey: A field study. J Comp Physiol A 197(5):403–412. 10.1007/s00359-010-0552-2 20582420

[msy192-B68] Shen Y-Y, Liu J, Irwin DM, Zhang Y-P. 2010 Parallel and convergent evolution of the dim-light vision gene RH1 in bats (Order: Chiroptera). PLoS One 5(1):e8838. 20098620 10.1371/journal.pone.0008838 PMC2809114

[msy192-B69] Shi Y, Radlwimmer FB, Yokoyama S. 2001 Molecular genetics and the evolution of ultraviolet vision in vertebrates. Proc Natl Acad Sci U S A. 98(20):11731–11736. 11573008 10.1073/pnas.201257398 PMC58798

[msy192-B70] Shimodaira H. 2002 An approximately unbiased test of phylogenetic tree selection. Syst. Biol. 51(3):492–508. 12079646 10.1080/10635150290069913

[msy192-B71] Shimodaira H, Hasegawa M. 2001 CONSEL: For assessing the confidence of phylogenetic tree selection. Bioinformatics 17(12):1246–1247. 11751242 10.1093/bioinformatics/17.12.1246

[msy192-B72] Sidell BD, O’Brien KM. 2006 When bad things happen to good fish: The loss of hemoglobin and myoglobin expression in Antarctic icefishes. J Exp Biol. 209(Pt 10):1791–1802. 16651546 10.1242/jeb.02091

[msy192-B73] Simmons NB. 2005 Order Chiroptera In: Wilson DE, Reeder DM, Baltimore, editors. Mammalian species of the world: A taxonomic and geographic reference. Baltimore: Johns Hopkins University Press.

[msy192-B74] Simões BF, Sampaio FL, Douglas RH, Kodandaramaiah U, Casewell NR, Harrison RA, Hart NS, Partridge JC, Hunt DM, Gower DJ. 2016 Visual pigments, ocular filters and the evolution of snake vision. Mol Biol Evol. 33(10):2483–2495. 27535583 10.1093/molbev/msw148

[msy192-B75] Simões BF, Sampaio FL, Jared C, Antoniazzi MM, Loew ER, Bowmaker JK, Rodriguez A, Hart NS, Hunt DM, Partridge JC, et al 2015 Visual system evolution and the nature of the ancestral snake. J Evol Biol. 28(7):1309–1320. 26012745 10.1111/jeb.12663

[msy192-B76] Smotherman M, Guillén-Servent A. 2008 Doppler-shift compensation behavior by Wagner’s mustached bat, *Pteronotus personatus*. J Acoust Soc Am. 123(6):4331–4339. 18537384 10.1121/1.2912436 PMC2680666

[msy192-B77] Springer MS, Emerling CA, Fugate N, Patel R, Starrett J, Morin PA, Hayashi C, Gatesy J. 2016 Inactivation of cone-specific phototransduction genes in rod monochromatic cetaceans. Front Ecol Evol. 4:61.

[msy192-B78] Stamatakis A. 2006 RAxML-VI-HPC: Maximum likelihood-based phylogenetic analyses with thousands of taxa and mixed models. Bioinformatics 22(21):2688–2690. 16928733 10.1093/bioinformatics/btl446

[msy192-B79] Stoffberg S, Jacobs DS, Mackie IJ, Matthee CA. 2010 Molecular phylogenetics and historical biogeography of *Rhinolophus* bats. Mol Phylogenet Evol. 54(1):1–9. 19766726 10.1016/j.ympev.2009.09.021

[msy192-B80] Sun K, Feng J, Zhang Z, Xu L, Liu Y. 2009 Cryptic diversity in Chinese rhinolophids and hipposiderids (Chiroptera: Rhinolophidae and Hipposideridae). Mammalia 73:135–141.

[msy192-B81] Tan Y, Yoder AD, Yamashita N, Li W-H. 2005 Evidence from opsin genes rejects nocturnality in ancestral primates. Proc Natl Acad Sci U S A. 102(41):14712–14716. 16192351 10.1073/pnas.0507042102 PMC1253590

[msy192-B82] Teeling CE, Scally M, Kao DJ, Romagnoli ML, Springer MS, Stanhope MJ. 2000 Molecular evidence regarding the origin of echolocation and flight in bats. Nature 403:188–192. 10646602 10.1038/35003188

[msy192-B83] Teeling E, Vernes S, Davalos LM, Ray DA, Gilbert MTP, Myers E, Consortium BK. 2018 Bat biology, genomes, and the Bat1K Project: To generate chromosome-level genomes for all living bat species. Annu Rev Anim Biosci. 6(12):11–12.24. 10.1146/annurev-animal-022516-022811 29166127

[msy192-B84] Teeling EC. 2009 Bats (Chiroptera) In: Hedges B, Kumar S, editors. The timetree of life. Oxford: Oxford University Press p. 499–503.

[msy192-B85] Teeling EC, Jones G, Rossiter SJ. 2016 Phylogeny, genes, and hearing: Implications for the evolution of echolocation in bats In: Fenton MB, Grinnell AD, Popper AN, Fay RR, editors. Bat bioacoustics. New York: Springer p. 25–54.

[msy192-B86] Teeling EC, Madsen O, Van Den Bussche RA, de Jong WW, Stanhope MJ, Springer MS. 2002 Microbat paraphyly and the convergent evolution of a key innovation in Old World rhinolophoid microbats. Proc Natl Acad Sci U S A. 99(3):1431–1436. 11805285 10.1073/pnas.022477199 PMC122208

[msy192-B87] Teeling EC, Springer MS, Madsen O, Bates P, O’Brien SJ, Murphy WJ. 2005 A molecular phylogeny for bats illuminates biogeography and the fossil record. Science 307(5709):580–584. 15681385 10.1126/science.1105113

[msy192-B89] Veilleux CC, Cummings ME. 2012 Nocturnal light environments and species ecology: implications for nocturnal color vision in forests. J Exp Biol. 215:4085–4096. 22899522 10.1242/jeb.071415

[msy192-B90] Veilleux CC, Louis EE Jr, Bolnick DA. 2013 Nocturnal light environments influence color vision and signatures of selection on the OPN1SW opsin gene in nocturnal lemurs. Mol Biol Evol. 30(6):1420–1437. 23519316 10.1093/molbev/mst058

[msy192-B92] Wang D, Oakley T, Mower J, Shimmin LC, Yim S, Honeycutt RL, Tsao H, Li W-H. 2004 Molecular evolution of bat color vision genes. Mol Biol Evol. 21(2):295–302. 14660703 10.1093/molbev/msh015

[msy192-B93] Winter Y, von Helversen O. 2003 Operational tongue length in phyllostomid nectar-feeding bats. J Mammal. 84(3):886–896.

[msy192-B94] Wu Y, Wang H, Hadly EA. 2017 Invasion of ancestral mammals into dim-light environments inferred from adaptive evolution of the phototransduction genes. Sci Rep. 7:1–9. 28425474 10.1038/srep46542 PMC5397851

[msy192-B95] Yang Z. 2007 PAML 4: Phylogenetic analysis by maximum likelihood. Mol Biol Evol. 24(8):1586–1591. 17483113 10.1093/molbev/msm088

[msy192-B96] Yang Z, Wong WS, Nielsen R. 2005 Bayes empirical Bayes inference of amino acid sites under positive selection. Mol Biol Evol. 22(4):1107–1118. 15689528 10.1093/molbev/msi097

[msy192-B97] Yokoyama S. 2000 Molecular evolution of vertebrate visual pigments. Prog. Retin. Eye Res. 19(4):385–419. 10785616 10.1016/s1350-9462(00)00002-1

[msy192-B98] Yokoyama S, Radlwimmer FB. 1998 The “five-sites” rule and the evolution of red and green color vision in mammals. Mol Biol Evol. 15(5):560–567. 9580985 10.1093/oxfordjournals.molbev.a025956

[msy192-B99] Yokoyama S, Shi Y. 2000 Genetics and evolution of ultraviolet vision in vertebrates. FEBS Lett. 486(2):167–172. 11113460 10.1016/s0014-5793(00)02269-9

[msy192-B100] Yokoyama S, Starmer WT, Takahashi Y, Tada T. 2006 Tertiary structure and spectral tuning of UV and violet pigments in vertebrates. Gene 365:95–103. 16343816 10.1016/j.gene.2005.09.028 PMC2810422

[msy192-B101] Yokoyama S, Yang H, Starmer WT. 2008 Molecular basis of spectral tuning in the red-and green-sensitive (M/LWS) pigments in vertebrates. Genetics 179(4):2037–2043. 18660543 10.1534/genetics.108.090449 PMC2516078

[msy192-B102] Yokoyama S, Yokoyama R. 1996 Adaptive evolution of photoreceptors and visual pigments in vertebrates. Annu Rev Ecol Evol Syst. 27(1):543–567.

[msy192-B150] Yokoyama S. 2008 Evolution of dim-light and color vision pigments. Annu Rev Genomics Hum Genet. 9:259–282. 18544031 10.1146/annurev.genom.9.081307.164228

[msy192-B104] Zhao H, Rossiter SJ, Teeling EC, Li C, Cotton JA, Zhang S. 2009 The evolution of color vision in nocturnal mammals. Proc Natl Acad Sci U S A. 106(22):8980–8985. 19470491 10.1073/pnas.0813201106 PMC2690009

[msy192-B105] Zhao H, Ru B, Teeling EC, Faulkes CG, Zhang S, Rossiter SJ. 2009 Rhodopsin molecular evolution in mammals inhabiting low light environments. PLoS One 4(12):e8326. 20016835 10.1371/journal.pone.0008326 PMC2790605

[msy192-B106] Zhao H, Yang J-R, Xu H, Zhang J. 2010 Pseudogenization of the umami taste receptor gene Tas1r1 in the giant panda coincided with its dietary switch to bamboo. Mol Biol Evol. 27(12):2669–2673. 20573776 10.1093/molbev/msq153 PMC3108379

