# Supplementary material for: As Blind as a Bat? Opsin Phylogenetics Illuminates the Evolution of Color Vision in Bats
Source: Mol Biol Evol. 2018 Nov 23;36(1):54–68. doi: 10.1093/molbev/msy192 (PMC6340466; doi:10.1093/molbev/msy192)
Supplement: Supplementary Data [file msy192_supp.zip › Simoes_etal_Final_Supplementary Information.pdf]

Supplementary Information

As Blind as a Bat? Opsin Phylogenetics Illuminates the Evolution of Color Vision in Bats.

Bruno F. Simões[ab], Nicole M. Foley[a], Graham M. Hughes[a], Huabin Zhao[c], Shuyi Zhang[d], Stephen J. Rossiter[e] and Emma C. Teeling[a,*]

[a] UCD School of Biology and Environmental Science, University College Dublin, Belfield, Dublin 4, Ireland;
[b] School of Earth Science, University of Bristol, Bristol, BS8 1TG, United Kingdom and School of Biological Science, The University of Adelaide, North Terrace, South Australia 5005, Australia.
[c] College of Life Sciences, Wuhan University, Wuhan, Hubei 430072, China
[d] College of Animal Science and Veterinary Medicine, Shenyang Agricultural University, Shenyang 110866,China.
[e] School of Biological and Chemical Sciences, Queen Mary University of London, London E1 4NS, United Kingdom

*Corresponding Author: E-mail: emma.teeling@ucd.ie

List of Figures:

Figure S1: Convergent phylogram inferred from ML analysis of species with functional *SWS1* genes only.
Figure S2: Time tree inferred from BEAST analysis of Cyt *b* dataset of species from the families Mormoopidae and Phyllostomidae.
Figure S3: Time tree inferred from BEAST analysis of Cyt *b* dataset to resolve relationships within the Megadermatidae.
Figure S4: Time tree inferred from BEAST analysis of Cyt *b* dataset of species from the family Pteropodidae
Figure S5: Species tree used for the PAML selection test analyses for the *SWS1* gene
Figure S6: Species tree used for the PAML selection test analyses for the *MWS/LWS* opsin gene.

List of Tables:

Table S1: Timing of the relaxation of functional constraint in lineages where the *SWS1* is pseudogenised.
Table S2: List of taxa included in this study.
Table S3: List of primers and primer sequences used as part of this study.
Table S4: Amino acid sites inferred to be under positive selection for SWS1 and MWS/LWS in bats.
Table S5: Brunch contrasts for the SWS1 opsin gene and primarily cave roosting habits in pteropodids.

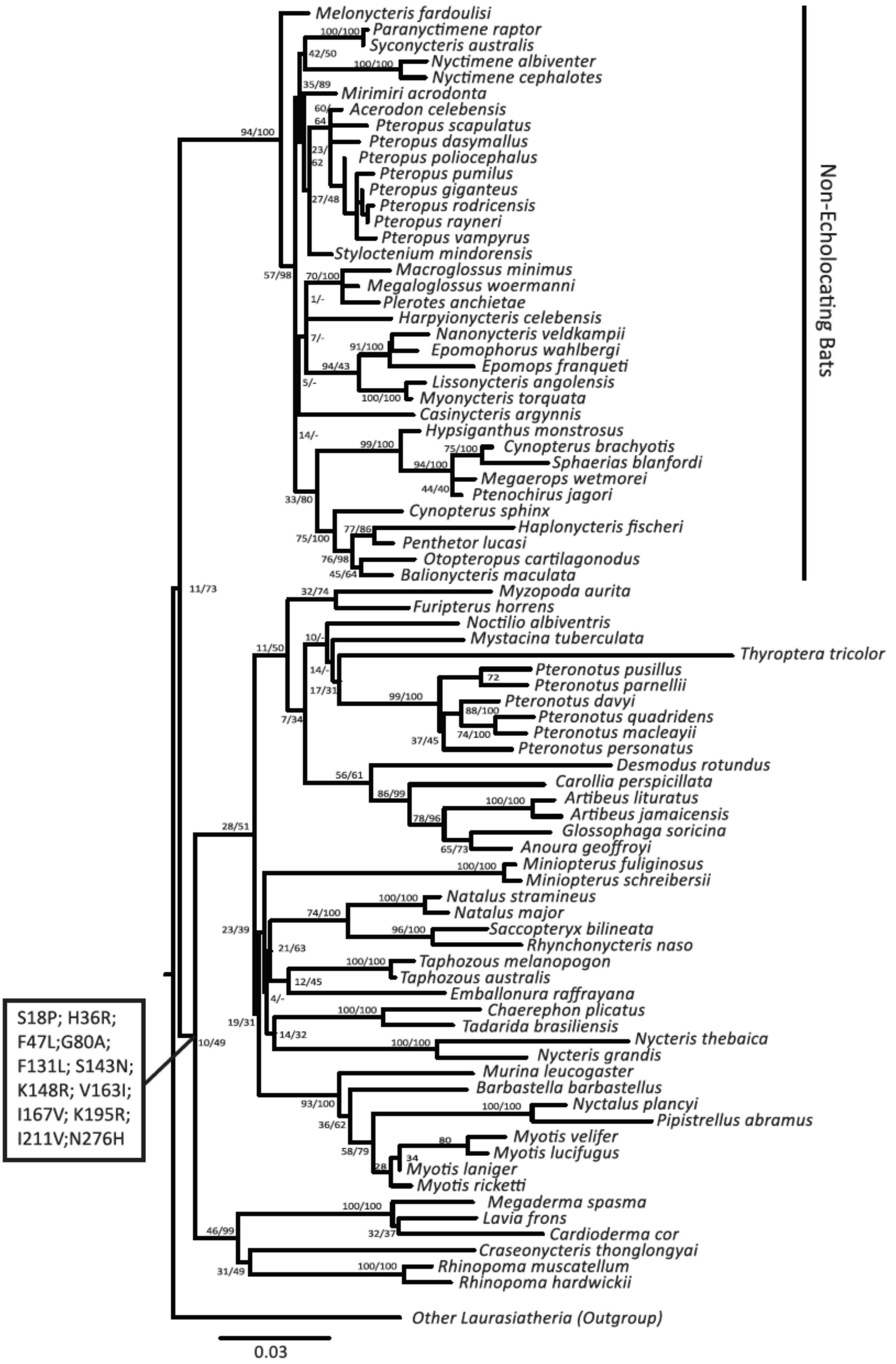

Figure S1: Convergent phylogram inferred from ML analysis of species with functional *SWS1* genes only. Nodal support for both the ML and BA analysis are shown at nodes corresponding to major clades. Ancestral state reconstruction of *SWS1* spectral tuning sites shown in the inset for the common ancestor of all echolocating bats

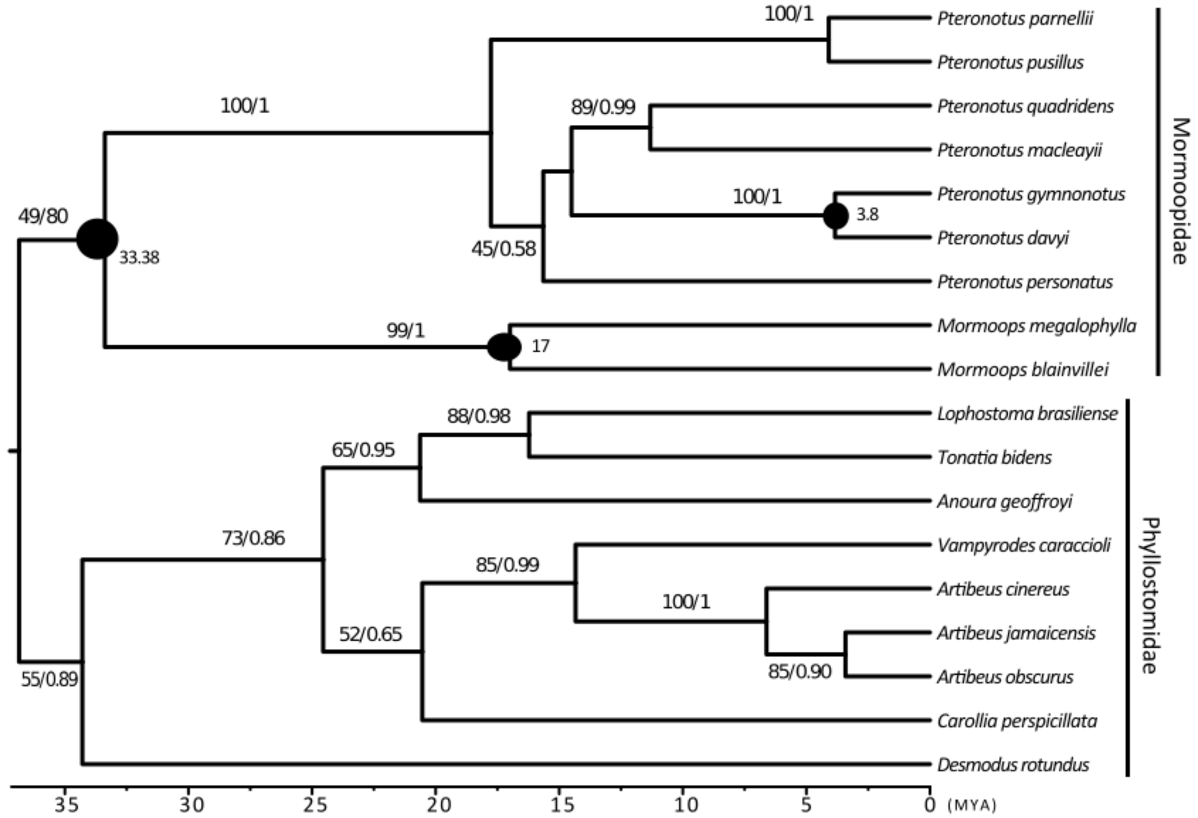

Figure S2: Time tree inferred from BEAST analysis of Cyt *b* dataset of species from the families Mormoopidae and Phyllostomidae. Bootstrap values and Bayesian posterior probabilities are shown at each node. Divergence estimates for clades which have undergone *SWS1* pseudogenisation (nodes marked with a black dot) are shown.

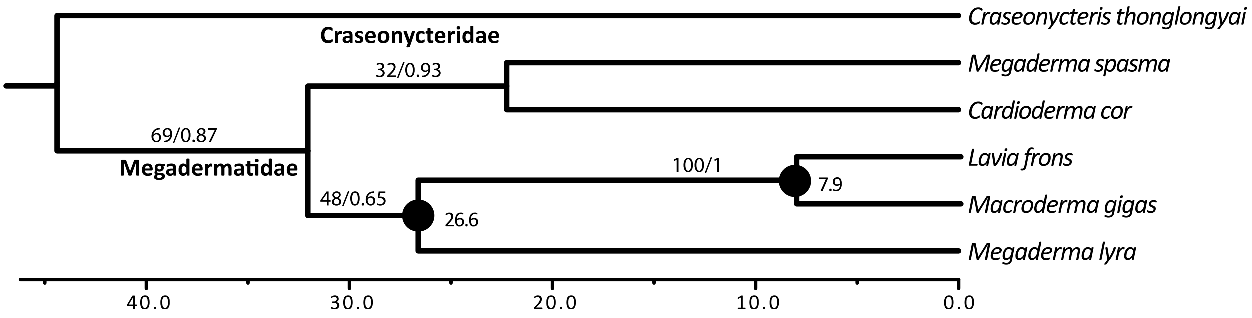

Figure S3: Time tree inferred from BEAST analysis of Cyt *b* dataset to resolve relationships within the Megadermatidae.Bootstrap support and Bayesian posterior probabilities are shown at each node. Divergence estimates for clades which have undergone *SWS1* pseudogenisation (nodes marked with a black dot) are shown.

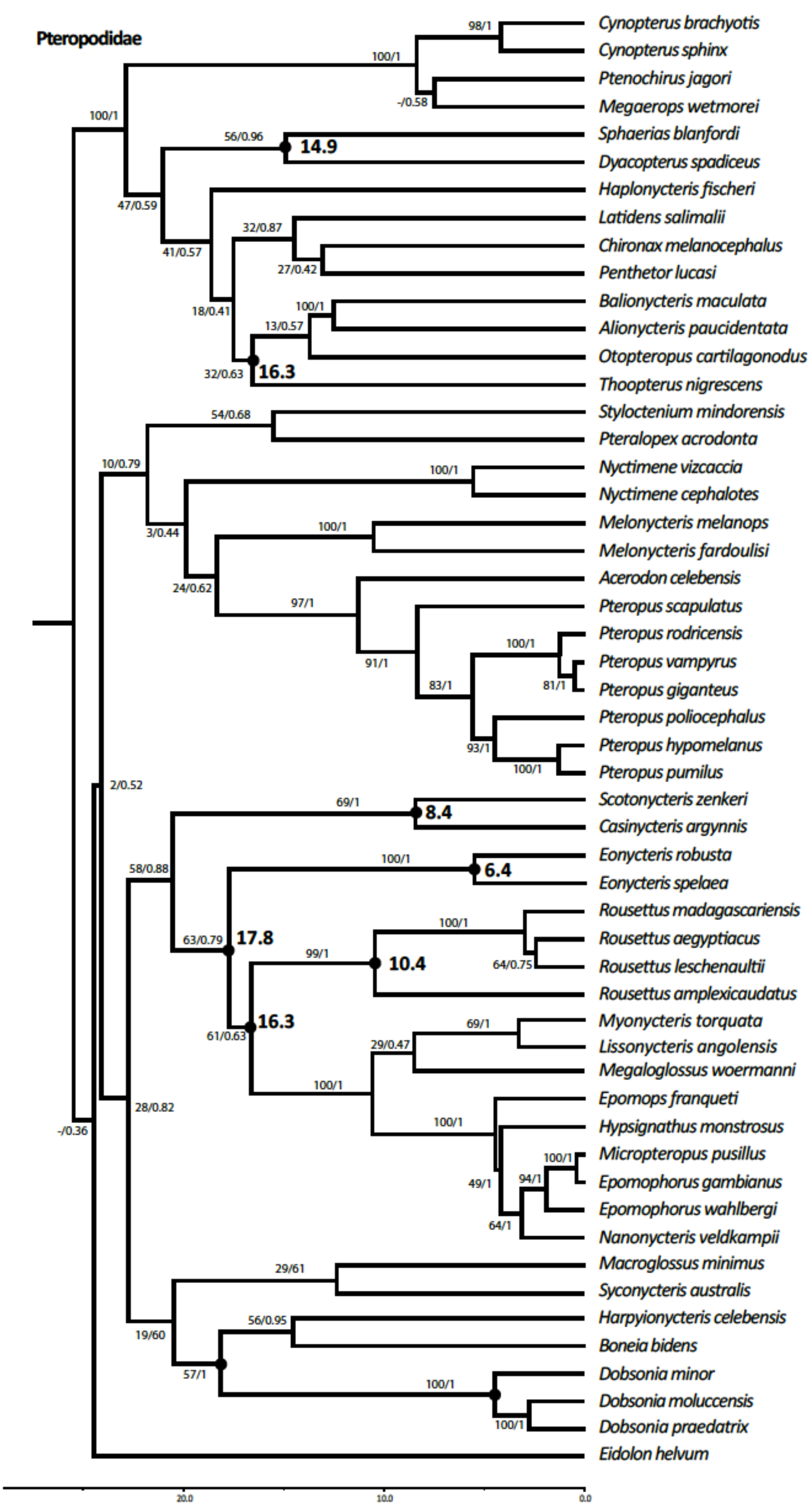

Figure S4: Time tree inferred from BEAST analysis of Cyt *b* dataset of species from the family Pteropodidae. Bootstrap support and Bayesian posterior probabilities are shown at each node. Divergence estimates for clades which have undergone *SWS1* pseudogenisation (nodes marked with a black dot) are shown

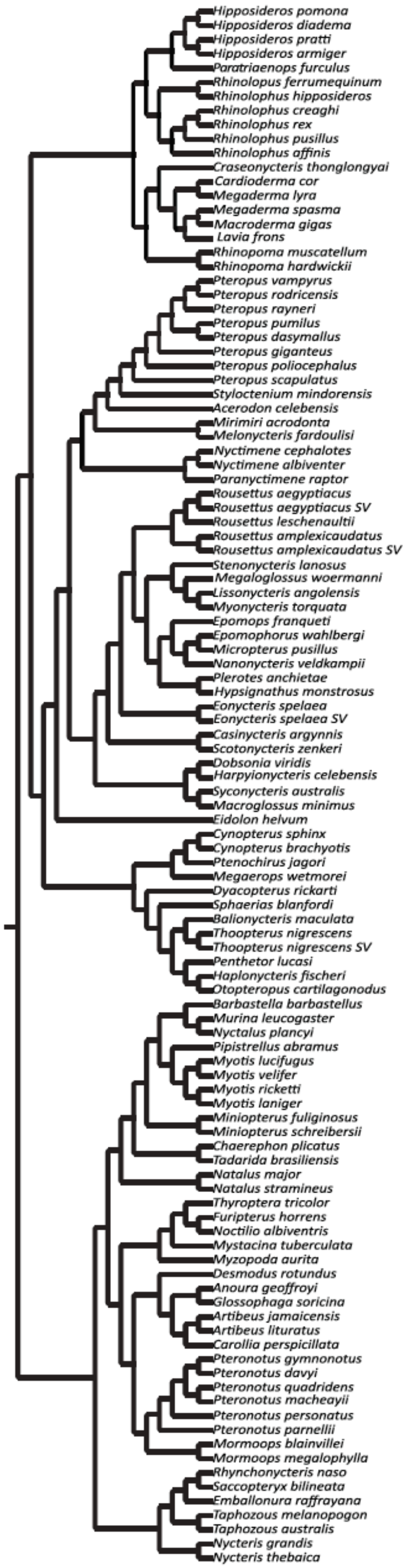

Figure S5: Species tree used for the PAML selection test analyses for the *SWS1* gene

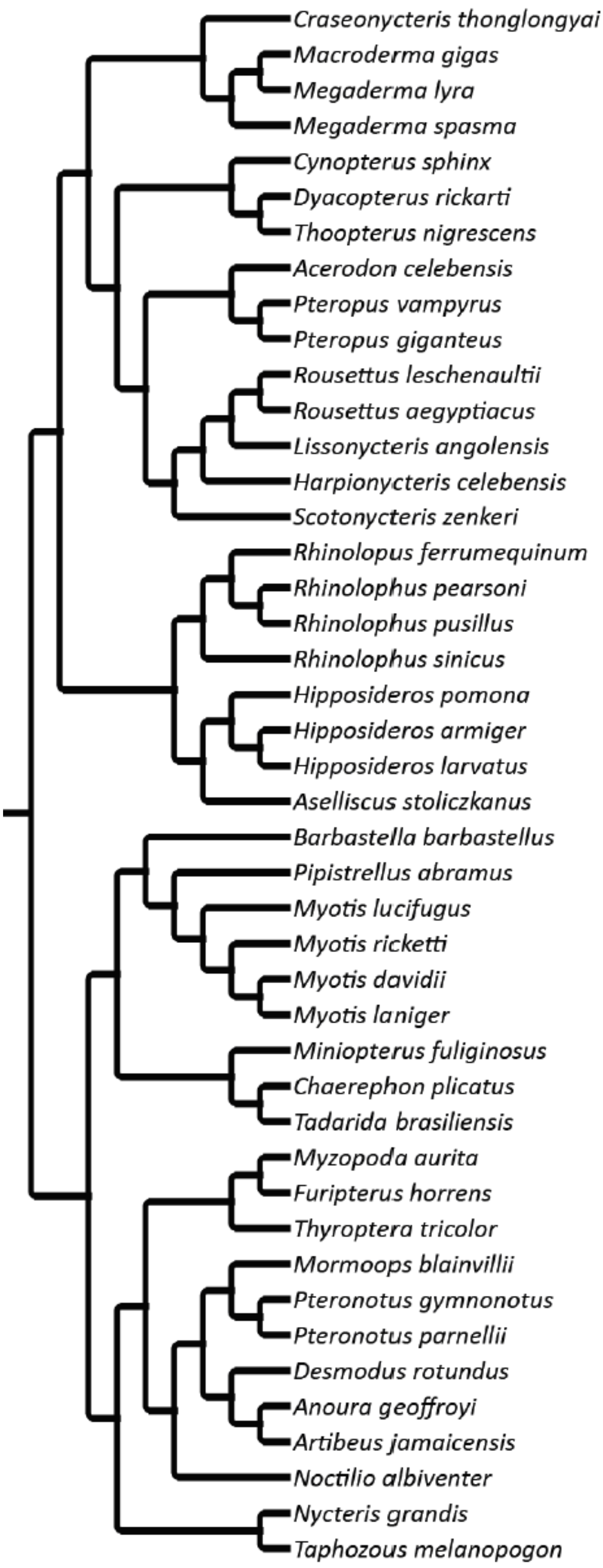

Figure S6: Species tree used for the PAML selection test analyses for the *MWS/LWS* opsin gene.

Lineage	Relaxation of the functional constrain according to:		
	**Zhao *et al.*, 2010**	**Meredith *et al.*, 2009**	**This study**
Rhinolophidae	29.1	31.35 – 27.11	42 – 17
Hipposideridae	28.4	31.35 – 27.11	42 – 15
Megaderma lyra	4.06	9.21 – 6.74	26.5 – 0.0
Macroderma gigas	2.86	5.82 – 2.70	7.8 – 0.0
Eidolon helvum	9.56	14.31 – 12.24	24.5 – 0.0
Rousettus	12.01	10.24 – 8.70	16.3 – 10.4
Dobsonia	6.79	11.37 – 8.44	18.1 – 4.6
Eonycteris	10.96	11.18 – 9.49	17.8 – 6.4
Scotonycteris	7.54	5.90 – 5.01	8.4 – 0.0
Dyacopterus	4.63	9.36 – 8.69	14.9 – 0.0
Thoopterus	11.32	10.24 – 8.69	16.3 – 0.0
Mormoops	29.78	26.23 – 24.09	33.3 – 17.0
Pteronotus gymnonotus	3.36	3.02 – 1.8	3.84 – 0.0

Table S1: Timing of the relaxation of functional constraint in lineages where the *SWS1* is pseudogenised. Estimates from this study are compared to estimates obtained form previously published studies.

Family	Species	Voucher ID	*SWS1*	*MWS/LWS*
**Ingroup: Laurasiatheria | Chiroptera**				
**Pteropodidae**	*Eidolon helvum*	–	EU912373	–
**Pteropodidae**	*Eonycteris spelaea*	–	EU912374	–
**Pteropodidae**	*Stenonycteris lanosus*	–	MH664114	–
**Pteropodidae**	*Rousettus amplexicaudatus*	–	EU912352	–
**Pteropodidae**	*Rousettus amplexicaudatus*	–	MH664113	–
**Pteropodidae**	*Rousettus leschenaultii*	–	EU912381	–
**Pteropodidae**	*Rousettus aegyptiacus*[(1)]	SMF87698 [(2)]	MH664112	MH392185
**Pteropodidae**	*Thoopterus nigrescens*	–	EU912355	–
**Pteropodidae**	*Thoopterus nigrescens*[(1)]	SFM93659 [(2)]	MH664122	MH392188
**Pteropodidae**	*Haplonycteris fischeri*	–	AM263192	–
**Pteropodidae**	*Nyctimene cephalotes*	–	EU912357	–
**Pteropodidae**	*Nyctimene albiventer*[(1)]	–	MH664088	–
**Pteropodidae**	*Dobsonia viridis*	–	EU912371	–
**Pteropodidae**	*Acerodon celebensis*	–	EU912354	EU912438
**Pteropodidae**	*Pteropus vampyrus*[(1)]	–	Ensemble v63	Ensemble v63
**Pteropodidae**	*Pteropus scapulatus*[(1)]	–	MH664105	–
**Pteropodidae**	*Pteropus poliocephalus*[(1)]	–	MH664104	–

**Family**	**Species**	**Voucher ID**	***SWS1***	***MWS/LWS***
**Pteropodidae**	*Pteropus rayneri*[(1)]	–	EU912397	EU912363
**Pteropodidae**	*Pteropus dasymallus*	–	AM263192	–
**Pteropodidae**	*Pteropus rodricensis*	–	EU912363	–
**Pteropodidae**	*Pteropus pumilus*	–	EU912362	–
**Pteropodidae**	*Pteropus giganteus*	–	EU912361	–
**Pteropodidae**	*Harpyionycteris celebensis*	–	EU912356	–
**Pteropodidae**	*Cynopterus brachyotis*	–	EU912353	–
**Pteropodidae**	*Cynopterus sphinx*	–	–	EU912342
**Pteropodidae**	*Cynopterus sphinx*[(1)]	–	MH664061	–
**Pteropodidae**	*Lissonycteris angolensis*[(1)]	NICD (SA)[(3)]	MH664070	MH392175
**Pteropodidae**	*Nanonycteris veldkampii*[(1)]	SMF92064 [(2)]	MH664084	–
**Pteropodidae**	*Hypsignathus monstrosus*[(1)]	SMF92969 [(2)]	MH664068	–
**Pteropodidae**	*Plerotes anchietae*[(1)]	SMF85745 [(2)]	MH664094	–
**Pteropodidae**	***Scotonycteris zenkeri***[(1)]	SMF92160 [(2)]	MH664116	MH392186
**Pteropodidae**	*Penthetor lucasi*[(1)]	SMF69266 [(2)]	MH664093	–
**Pteropodidae**	*Balionycteris maculata*[(1)]	USNM597672 [(4)]	MH664056	–
**Pteropodidae**	*Paranyctimene raptor*[(1)]	USNM585681 [(4)]	MH664092	–
**Pteropodidae**	*Syconycteris australis*[(1)]	USNM 585698 [(4)]	MH664119	–

Family	Species	Voucher ID	*SWS1*	*MWS/LWS*
**Pteropodidae**	*Dyacopterus rickarti*[1]	FMNH189976 [5]	MH664063	MH392173
**Pteropodidae**	*Epomophorus wahlbergi*[1]	FMNH198037 [5]	MH664064	–
**Pteropodidae**	*Megaerops wetmorei*[1]	FMNH206098 [5]	MH664074	–
**Pteropodidae**	*Otopteropus cartilagonodus*[1]	FMNH168998 [5]	MH664091	–
**Pteropodidae**	*Ptenochirus jagori*	FMNH169004 [5]	MH664095	–
**Pteropodidae**	*Styloctenium mindorensis*	FMNH190695 [5]	MH664118	–
**Pteropodidae**	*Sphaerias blanfordi*[1]	AMNH274201	MH664117	–
**Pteropodidae**		AMCC101547 [6]		
**Pteropodidae**	*Epomops franqueti*[1]	AMNH269902	MH664065	–
**Pteropodidae**		AMCC10943 [6]		
**Pteropodidae**	*Casinycteris argynnis*[1]	AMNH269902	MH664059	–
**Pteropodidae**		AMCC109046 [6]		
**Pteropodidae**	*Myonycteris torquata*[1]	AMNH268362	MH664081	–
**Pteropodidae**		AMCC109058 [6]		
**Pteropodidae**	*Megaloglossus woermanni*[1]	AMNH268360	MH664075	–
**Pteropodidae**		AMCC109064 [6]		
**Pteropodidae**	*Melonycteris fardoulisi*[1]	AMCC124279 [6]	MH664076	–
**Pteropodidae**	*Macroglossus minimus*[1]	AMCC124339 [6]	MH664072	–

**Family**	**Species**	**Voucher ID**	***SWS1***	***MWS/LWS***
**Pteropodidae**	*Mirimiri acrodonta*[(1)]	EBU9655 [(7)]	MH664078	–
**Rhinolophidae**	*Rhinolophus creaghi*	–	MH664106	–
**Rhinolophidae**	*Rhinolophus hipposideros*	–	MH664107-8	–
**Rhinolophidae**	*Rhinolophus rex*	–	EU912365	–
**Rhinolophidae**	*Rhinolophus affinis*	–	EU912364	–
**Rhinolophidae**	*Rhinolophus pusillus*	–	EU912382	–
**Rhinolophidae**	*Rhinolophus ferrumequinum*	–	EU912380	EU912349
**Hipposideridae**	*Hipposideros pratti*	–	EU912369	–
**Hipposideridae**	*Hipposideros armiger*	–	EU912368	EU912343
**Hipposideridae**	*Hipposideros pomona*	–	EU912369	–
**Hipposideridae**	*Hipposideros diadema*	–	MH664067	–
**Hipposideridae**	*Hipposideros larvatus*	–	–	GQ863445
**Hipposideridae**	*Aselliscus stoliczkanus*	AMCC125161[(6)]	–	MH392175
**Rhinonycteridae**	*Paratriaenops furculus*	–	MH664122	–
**Megadermatidae**	***Megaderma lyra***[(1)]	–	MH664073	MH392177
**Megadermatidae**	***Megaderma spasma***	–	EU912378	EU912346
**Megadermatidae**	***Macroderma gigas***[(1)]	–	MH664071	MH392176
**Megadermatidae**	***Lavia frons***	FMNH187138 [(5)]	MH664069	–

Family	Species	Voucher ID	*SWS1*	*MWS/LWS*
**Megadermatidae**	***Cardioderma cor***[1]	FMNH158010 [5]	MH664058	–
**Craseonycteridae**	*Craseonycteris thonglongyai*	–	MH664060	MH392171
**Rhinopomatidae**	*Rhinopoma hardwickii*	–	MH664109	–
**Rhinopomatidae**	*Rhinopoma muscatellum*	–	MH664110	–
**Vespertilionidae**	*Myotis lucifugus*[1]	–	Ensemble v63	Ensemble v63
**Vespertilionidae**	*Myotis ricketti*	–	EU912377	EU912345
**Vespertilionidae**	*Myotis velifer*	–	AM263197	GQ863441
**Vespertilionidae**	*Myotis laniger*	–	GQ863407	–
**Vespertilionidae**	*Pipistrellus abramus*	–	EU912360	EU912347
**Vespertilionidae**	*Murina leucogaster*	–	EU912379	–
**Vespertilionidae**	*Nyctalus plancyi*	–	GQ863408	–
**Vespertilionidae**	*Barbastella barbastellus*	–	MH664057	MH392170
**Miniopteridae**	*Miniopterus fuliginosus*	–	EU912376	EU912344
**Miniopteridae**	*Miniopterus schreibersii*	–	MH664077	MH392178
**Molossidae**	*Chaerephon plicatus*	–	EU912359	EU912341
**Molossidae**	*Tadarida brasiliensis*	–	MH664120	MH392187
**Nycteridae**	*Nycteris thebaica*	–	MH664090	–
**Nycteridae**	*Nycteris grandis*	–	MH664089	MH392182

Family	Species	Voucher ID	*SWS1*	*MWS/LWS*
**Emballonuridae**	*Taphozous melanopogon*	–	EU912382	EU912340
**Emballonuridae**	*Taphozous australis*	–	EU912383	–
**Emballonuridae**	*Emballonura raffrayana*	–	EU912374	–
**Emballonuridae**	*Rhynchonycteris naso*	–	MH664111	–
**Emballonuridae**	*Saccopteryx bilineata*	–	MH664115	–
**Phyllostomidae**	*Artibeus jamaicensis*	–	EU912367	EU912340
**Phyllostomidae**	*Artibeus lituratus*	–	EU912372	–
**Phyllostomidae**	*Carollia perspicillata*	–	EU912358	–
**Phyllostomidae**	*Anoura geoffroyi*	–	MH664055	MH392169
**Phyllostomidae**	*Desmodus rotundus*	–	MH664062	MH392172
**Phyllostomidae**	*Glossophaga soricina*	–	FJ815442	–
**Mormoopidae**	*Mormoops megalophylla*	ROM98443[(8)]	MH664080	–
**Mormoopidae**	*Mormoops blainvillei*[(1)]	AMCC102762[(6)]	MH664079	MH392179
**Mormoopidae**	*Pteronotus parnellii*[(1)]	ROM101360[(8)]	MH664100	MH392184
**Mormoopidae**	*Pteronotus gymnonotus*[(1)]	ROM104265[(8)]	MH664097-8	MH392183
**Mormoopidae**	*Pteronotus davyi*	ROM98436[(8)]	MH664096	–
**Mormoopidae**	*Pteronotus personatus*	ROM98438[(8)]	MH664101	–
**Mormoopidae**	***Pteronotus pusillus***	AMCC138110[(6)]	MH664102	–

Family	Species	Voucher ID	*SWS1*	*MWS/LWS*
**Mormoopidae**	*Pteronotus quadridens*	AMCC138132[6]	MH664103	–
**Mormoopidae**	*Pteronotus macleayii*	AMCC102674[6]	MH664099	
**Furipteridae**	*Furipterus horrens*	–	MH664066	MH392174
**Mystacinidae**	*Mystacina tuberculata*	–	MH664082	–
**Myzopodidae**	*Myzopoda aurita*	–	MH664083	MH392180
**Noctilionidae**	*Noctilio albiventris*	–	MH664087	MH392181
**Natalidae**	*Natalus stramineus*	–	MH664086	–
**Natalidae**	*Natalus major*	AMCC138128[6]	MH664085	–
**Thyropteridae**	*Thyroptera tricolor*	–	MH664121	MH392189
**Outgroup: Laurasiatheria | Cetartiodactyla, Carnivora, Perissodactyla and Eulipotypha**				
**Bovidae**	*Bos taurus*	–	NM_174567	NM_174566
**Bovidae**	*Tragelaphus eurycerus*	–	GU246413	-
**Camelidae**	*Vicugna pacos*	–	Ensemble v63	Ensemble v63
**Suidae**	*Sus scrofa*	–	AY091587	AY693774
**Delphinidae**	*Tursiops truncatus*	–	Ensemble v63	Ensemble v63
**Canidae**	*Canis lupus*	–	XM_539386	XM_538203
**Felidae**	*Felis catus*	–	Ensemble v63	Ensemble v63
**Ursidae**	*Ailuropoda melanoleuca*	–	Ensemble v63	Ensemble v63

**Family**	**Species**	**Voucher ID**	***SWS1***	***MWS/LWS***
**Equidae**	*Equus caballus*	–	XM_001502735	AF132043
**Erinaceidae**	*Erinaceus europaeus*	–	Ensemble v63	-
**Soricidae**	*Sorex aranaeus*	–	-	Ensemble v63

Table S2: List of taxa included in this study. Where available, voucher IDs are provided for specimens used in this study. Genbank accession numbers for novel sequence data generated as part of this study are highlighted in red. Species for which Cyt b was sequenced to determine the phylogenetic relationships and divergence time estimates are shown in bold. (1) Samples where the full length SWS1 opsin gene was sequenced. Samples were donated by: (2) Senckenberg Natural History Museum, Germany; (3) National Institute of Communicable Diseases, South Africa; (4) Smithsonian Institution National Museum of Natural History, USA; (5) Field Museum of Natural History of Chicago, USA; (6) Ambrose Monnel Cryogenic Collection – American Museum of Natural History; (7) Australian Museum of Natural History, Australia; (8) Royal Ontario Museum, Canada

Primer	Sequence	Reference
**SDC1R**	TATAGKACTCGCTGCGRTAYTTGGTGCC	Zhao *et al.*, 2009a
**SDR1F**	GCAGTGTTCCTGTGGYCCYGACTGGTAC	Zhao *et al.*, 2009a
**SFE**	ATGTCAGRGGARGAGTTTTATCTGTTCAAG	Zhao *et al.*, 2009a
**SINRb**	GGAACTGCTTATTCATGAAGCAGTAGATGATGGG	Zhao *et al.*, 2009a
**SWS_F0**	GTCGGGGGAGGGRTAATCTATAAGAG	This study
**SWS_F1**	GGGAGGAGGAGTTTTATCTGT	This study
**SWS_F2**	TAACAGGCTGGTCACTGG	This study
**SWS_F3**	AGGGCCTGCAATGTTCCT	This study
**SWS_F4**	CACCATTCCTGCCTTCTTCTCCA	This study
**SWS_R1**	AAAGAAGGGTGGGATGGA	This study
**SWS_R2**	CCCCCAGCAACTGAGAGTAG	This study
**SWS_R3**	AAGCAGTAGATGATGGGATTG	This study
**SWS_R4**	CTTAGCTGGGGCCCACTTGGCT	This study
**ChiF1 (COI)**	ATACTTCGGGGTGGCCGAAGAATCA	Boston *et al.*, 2010
**ChiR1 (COI)**	TYTCAACCAAYCACAAAGATATYGG	Boston *et al.*, 2010
**MtF2 (Cyt *b*)**	ATGGCCCYGAAGAAAGAACCAGATG	Boston *et al.*, 2010
**MtR3 (Cyt *b*)**	TGGCATGAAAAATCACCGTCT	Boston *et al.*, 2010

Table S3: List of primers and primer sequences used as part of this study.

Models	Sites Under Positive Selection
***SWS1* opsin gene - Chiroptera**	
B. M2a	46
D. M8 (β&ω)	46 – 135 – 278 – 330 – 338
***MWS/LWS* opsin gene**	
F. M2a	162 – 171 – **180** – 235
H. M8 (β&ω)	162 – 171 – **180** – 235
***SWS1* opsin gene - Pteropodidae**	
J. M2a	1 – 46 – 117 – 135
L. M8 (β&ω)	1 – 46 – 66 – 117 – 135 – 153 – 278

Table S4**.** Amino acid sites inferred to be under positive selection (using Bayes Empirical Bayes), identified under site models for the SWS1 and MWS/LWS in bat datasets. In bold are amino-acid sites known to impact spectral sensitivity. The amino acid positions refer to *Myotis lucifugus* SWS1 sequence (Chiroptera dataset) and *Pteropus vampyrus* SWS1 sequence (in the dataset Pteropodidae). The amino acid positions for the MWS/LWS refers to full length MWS/LWS opsin in *Myotis lucifugus.*

	SWS1	Cave.Roosters	contrVar	nChild	nodeDepth	nodeAge	studentResid
**67**	0.04454158	1	60.70396	2	4	19	-0.1269893
**72**	0.03101734	1	33.30864	2	2	17	-0.2463777
**48**	0.01935588	1	26.69156	2	2	25	-0.2475617
**84**	0.02035545	1	6.674654	2	2	2	-0.0475617
**97**	0.01935342	1	5.874531	2	2	5	-0.0394931

Table S5. Output of caic.table for the brunch contrasts for the SWS1 opsin gene and primarily cave roosting habits in the pteropodids.

References

Boston ES, Buckley DJ, Bekaert M, Gager Y, Lundy MG, Scott DD, Prodöhl PA, Montgomery WI, Marnell F, Teeling EC. 2010. The status of the cryptic bat species, *Myotis mystacinus* and *Myotis brandtii* in Ireland. *Acta Chiropterol.* 12:457-461.

Meredith RW, Gatesy J, Murphy WJ, Ryder OA, Springer MS. 2009. Molecular decay of the tooth gene enamelin (*ENAM*) mirrors the loss of enamel in the fossil record of placental mammals. *PLoS Genet.* 5:e1000634.

Zhao H, Rossiter SJ, Teeling EC, Li C, Cotton JA, Zhang S. 2009. The evolution of color vision in nocturnal mammals. *Proc Natl Acad Sci USA.*106:8980-8985.

Zhao H, Yang J-R, Xu H, Zhang J. 2010. Pseudogenization of the umami taste receptor gene *Tas1r1* in the giant panda coincided with its dietary switch to bamboo. *Mol Biol Evol.* 27:2669-2673.
