# Supplementary figures and images for: As Blind as a Bat? Opsin Phylogenetics Illuminates the Evolution of Color Vision in Bats
Source: Mol Biol Evol. 2018 Nov 23;36(1):54–68. doi: 10.1093/molbev/msy192 (PMC6340466; doi:10.1093/molbev/msy192)

# Non-Echolocating Bats

# Echolocating Bats

S18P; H36R;  
F47L;G80A;  
F131L; S143N;  
K148R; V163I;  
I167V; K195R;  
I211V;N276H

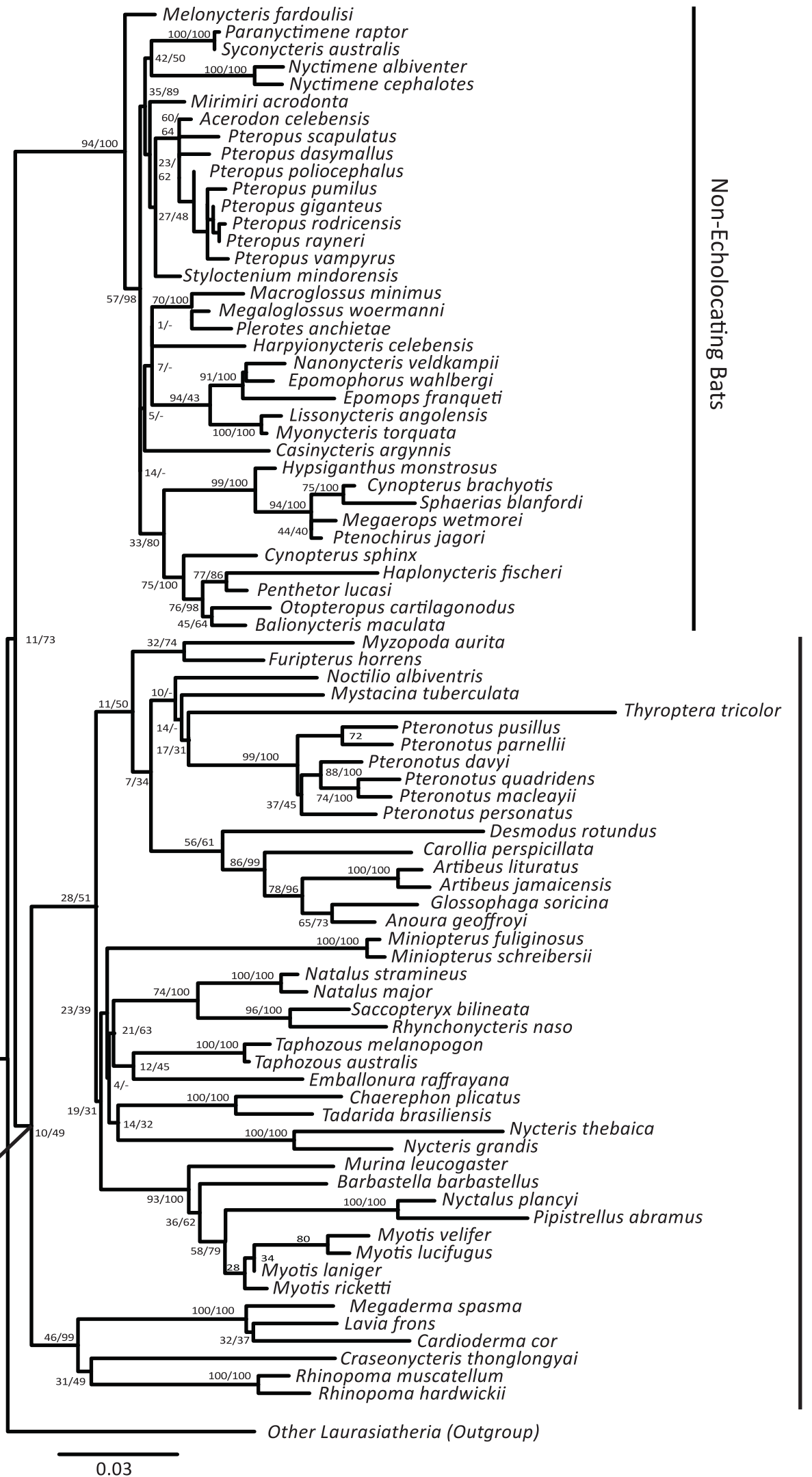

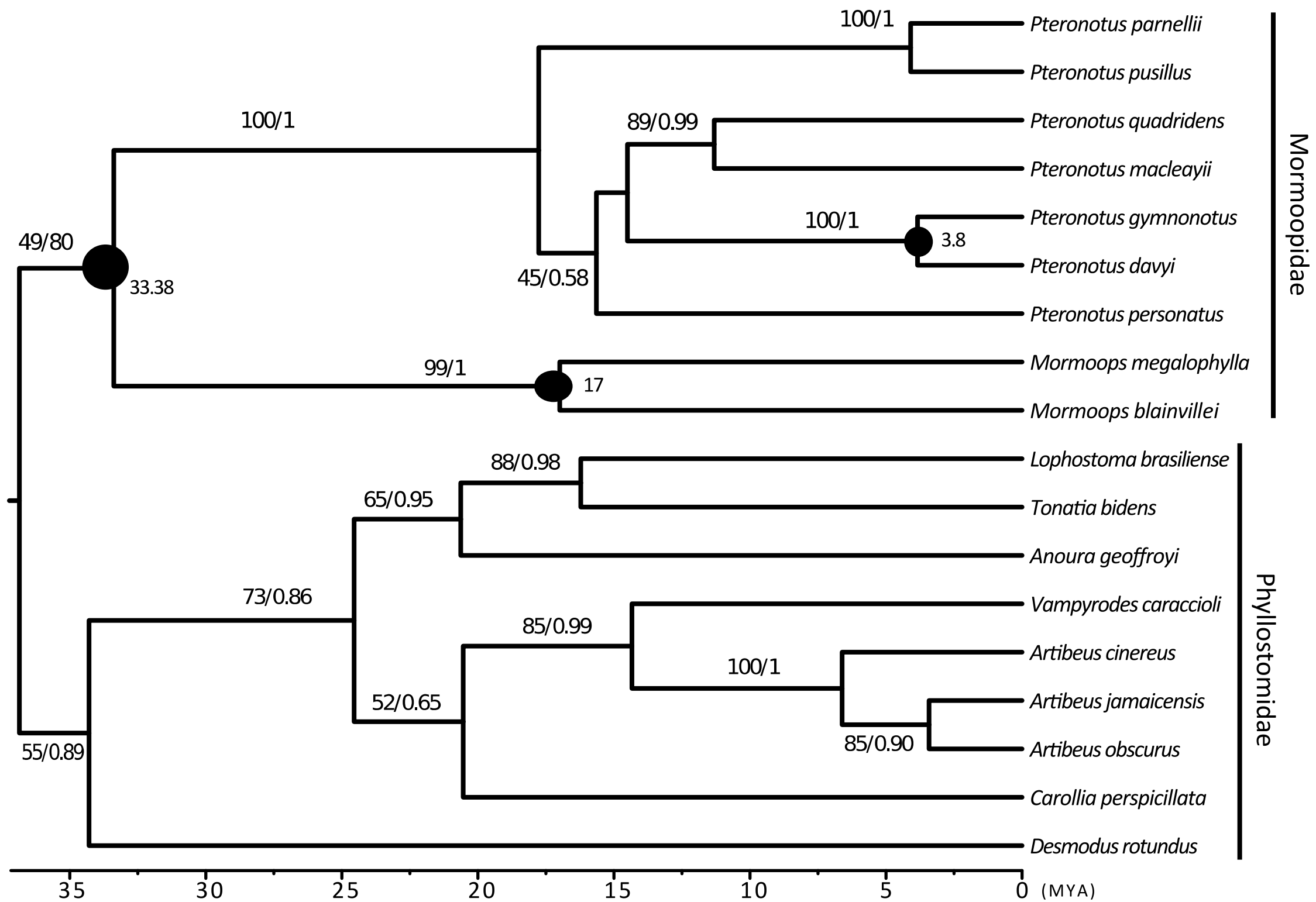

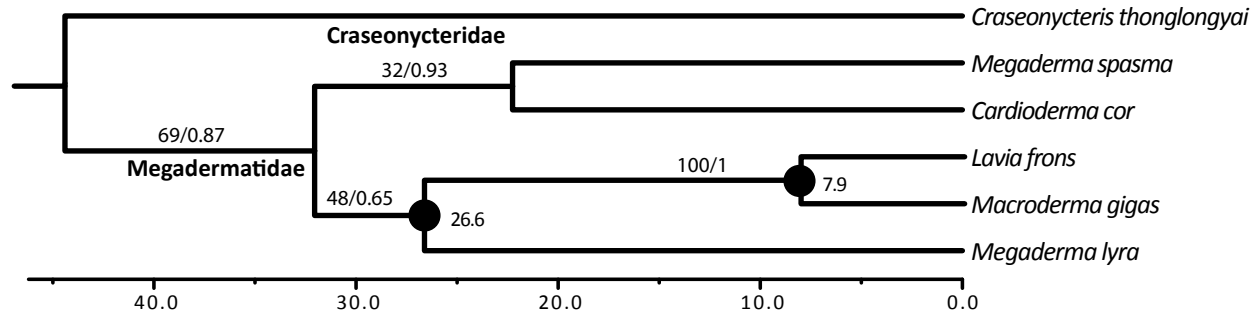

Pteropodidae

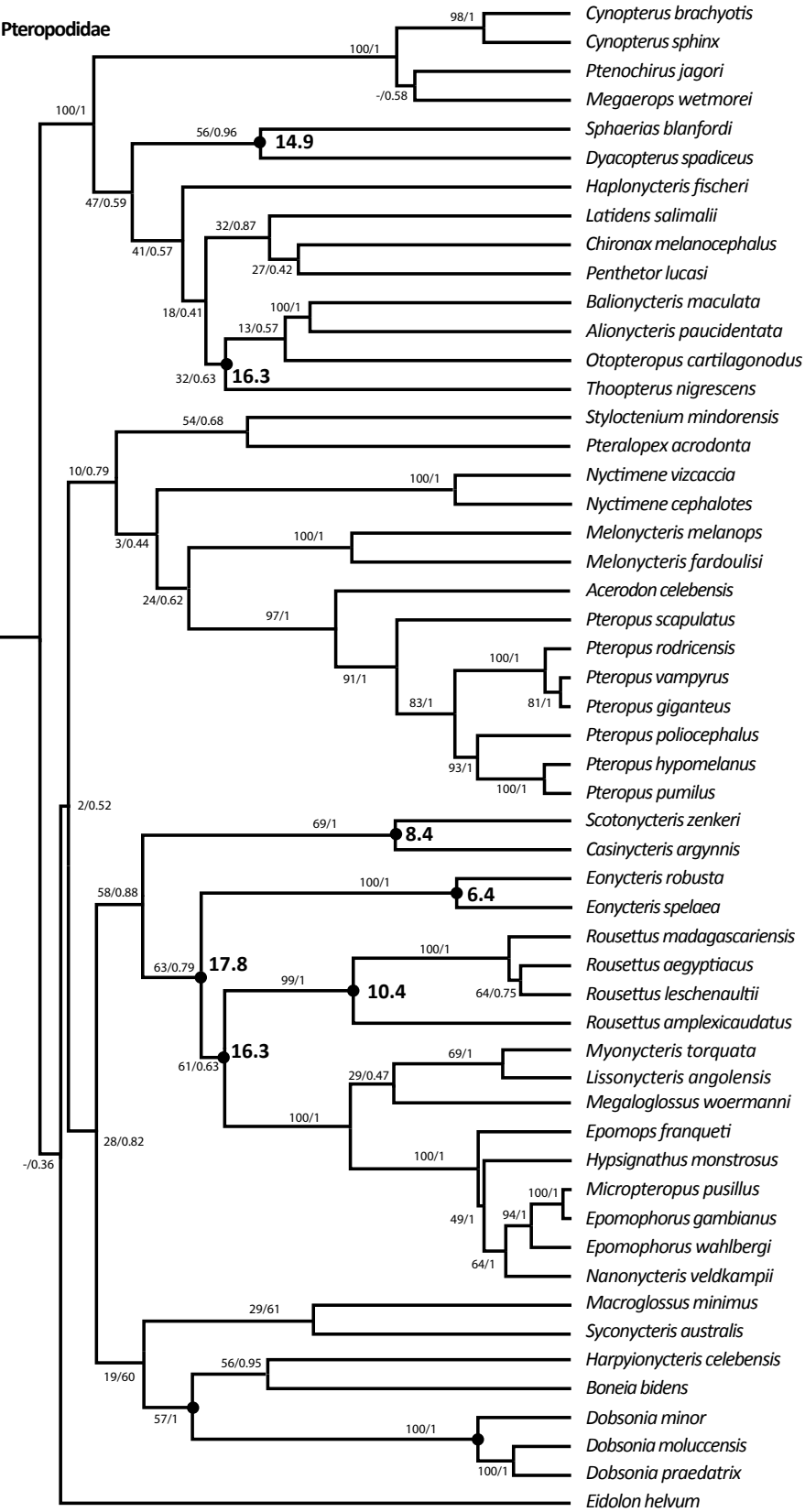

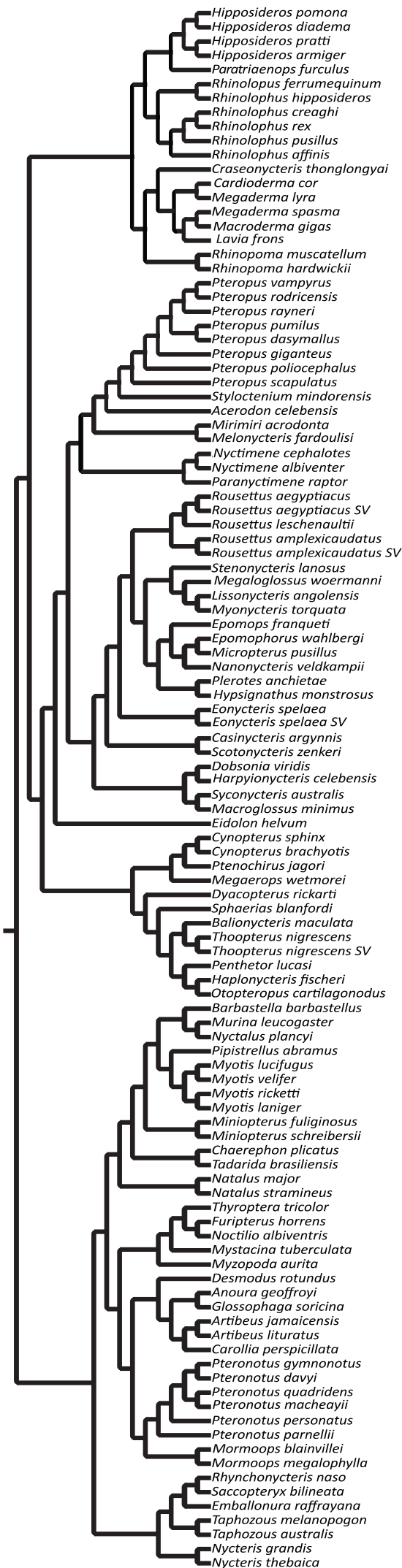

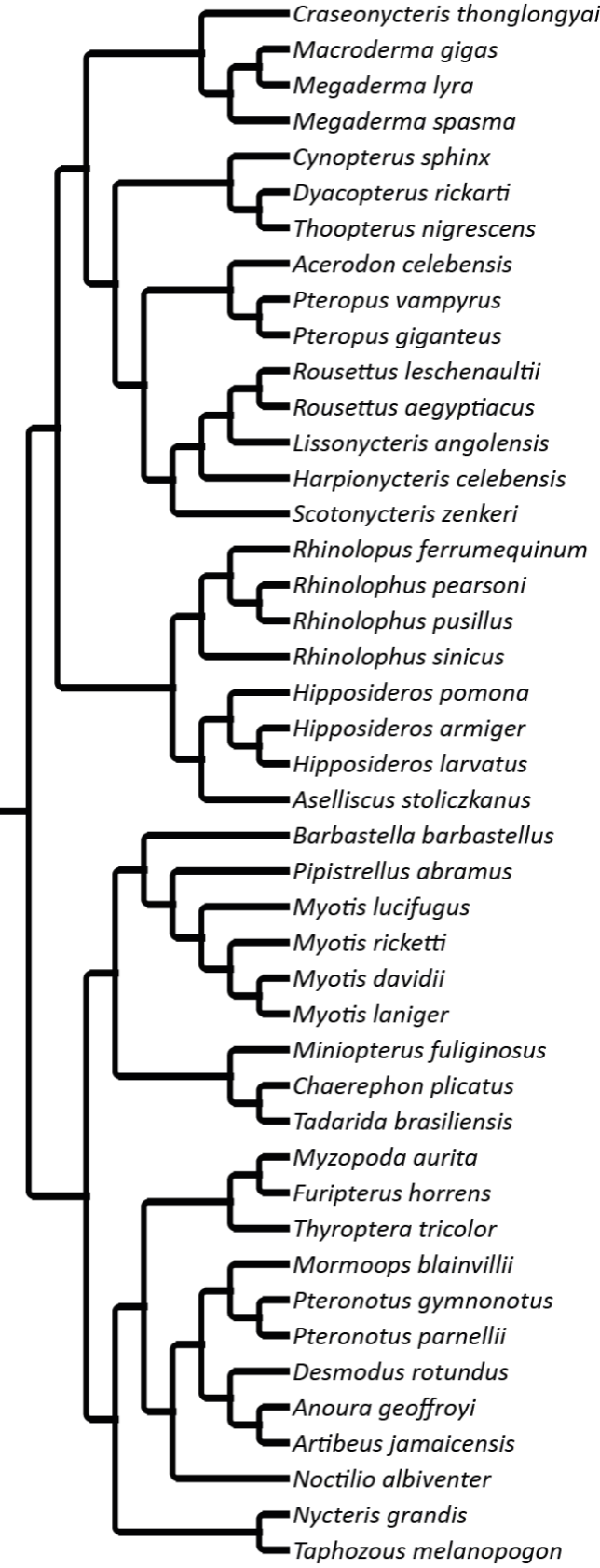

Supplement: Supplementary Data [file msy192_supp.zip › AllSupplementalFigures.pdf]
